# Supplementary material for: BN-Embedded Perylenes
Source: Molecules. 2021 Nov 25;26(23):7148. doi: 10.3390/molecules26237148 (PMC8658878; doi:10.3390/molecules26237148)
Supplement: Supplementary file 1 [file molecules-26-07148-s001.zip › molecules-1430771-SI.pdf]

Supporting Information

BN-Embedded Perylenes

Xiangdong Fang*

College of Chemical Science and Engineering, Tongji University, 1239 Siping Road, Yangpu, Shanghai 200092, China.
Correspondence: xdfang@tongji.edu.cn

Table of Contents

I. General Information……………………………………………………………S-2
II. Synthesis of **4**………………………..…………………………………………S-3
III. Synthesis of BN-Perylene **1**...…………………………….…………………S-3
IV. Synthesis of $(BN)_2$-Perylene **2** ………………………………………...……S-4
V. Determination of Structures of **1**, **2**, and **4**…………………………………..S-4
VI. DFT Calculations…………………………………………………………………S-9

NMR Spectra (^{1}H, ^{13}C, ^{11}B, COSY and HSQC) and HRMS

I. General Information

All air- and moisture-sensitive reactions were conducted with magnetic stirring in oven-dry glassware under nitrogen atmosphere using anhydrous solvents and standard Schlenk-line techniques with an MBraun Labstar-MB10 glovebox. The oxygen and moisture levels in the MBraun glovebox were constantly monitored by both oxygen and moisture analyzers to ensure O_2/H_2O levels below 0.1 ppm. Diethyl ether, tetrahydrofuran, and hexane were dried and distilled by treatment with sodium metal slices under a nitrogen atmosphere. Dichloromethane was dried and distilled by treatment with calcium hydride under a nitrogen atmosphere. Chemicals were purchased from chemical suppliers and used without further purification unless otherwise specified. Flash or thin-layer column chromatography was performed over silica gel (40-60 μm) purchased from Yantai Jiangyou Co., China.

^{1}H, ^{13}C, ^{11}B NMR spectra were collected on a Bruker ARX400 400 MHz NMR spectrometer using residue solvent peaks as an internal standard (^{1}H NMR: $CDCl_3$ at δ 7.26 ppm; ^{13}C NMR: $CDCl_3$ at δ 77.00 ppm; ^{11}B NMR: $BF_3{\cdot}Et_2O$ at δ 0.0 ppm). Data for ^{1}H NMR were recorded as follows: chemical shift (δ, ppm), multiplicity (s = singlet; d = doublet; t = triplet; m = multiplet; bs = broad singlet), coupling constant (*J* in Hz), integration. Mass spectra were collected at Bruker microTOF II ESI-TOF and Waters Micromass GCT Premier-TOF mass spectrometers. The UV-Vis and fluorescence spectra were collected on an Agilent 8354 and a Hitachi F-7000 spectrophotometer respectively. The X-ray single-crystal diffraction experiments were performed with a Bruker D8 VENTURE X-ray diffractometer with CMOS detector.

II. Synthesis of 4-bromo-5-(1-naphathyl)-*4a,8a*-azaboranaphthalene (4)

4,5-dibromo-*4a,8a*-azaboranaphthalene **3b** (28.6 mg, 0.1 mmol), 1-naphthylboronic acid (34.4 mg, 0.2 mmol), K_2CO_3 (27.7 mg, 0.2 mmol), and Pd(dppf)Cl_2 (3.6 mg, 0.005 mmol) were loaded in a Schlenk flask, which was then evacuated and recharged with nitrogen in three times. THF (3 mL) was injected with a syringe. The mixture was heated 12 hr at 110 °C. The solvent was removed by vacuum and the residue was extracted with CH_2Cl_2/H_2O twice. The combined organic layers were washed with brine and dried with anhydrous $MgSO_4$. The solution was concentrated and further purified by flash column chromatography (hexane:dichloromethane = 10:1). Product **4** was obtained as a light-yellow solid (37.3 mg, 56%).

^{1}H NMR (400 MHz, $CDCl_3$): δ 7.97-7.79 (m, 5H), 7.64-7.51 (m, 3H), 7.46 (t, *J* = 7.0 Hz, 1H), 7.32 (t, *J* = 6.3 Hz, 2H), 6.90 (t, *J* = 6.9 Hz, 1H), 6.59 (t, *J* = 7.1 Hz, 1H).

^{13}C NMR (101 MHz, $CDCl_3$): δ 143.2, 141.8, 140.2, 134.1, 133.8, 133.4, 126.7, 126.2, 125.4, 114.3, 113.9.

^{11}B NMR (193 MHz, $CDCl_3$): δ 26.99.

HRMS (EI+, 70 eV): calculated for $C_{18}H_{13}{}^{10}BBrN$ $[M]^+$: 332.0361; found: 332.0370.

III. Synthesis of *3a*-aza-*9a*-bora-perylene (1)

4-bromo-5-(1-naphthyl)-*4a,8a*-azaboranaphthalene (**4**) (33.4 mg, 0.1 mmol), LiCl (4.3 mg, 0.1 mmol), K_2CO_3 (27.6 mg, 0.2 mmol), Pd(OAc)$_2$ (2.3 mg, 0.005 mmol), and Xantphos (5.8 mg, 0.01 mmol) were loaded in a Schlenk flask, which was then evacuated and recharged with nitrogen three times. 1,4-Dioxane (5 mL) was injected with a syringe. The mixture was heated for 12 hr at 130 °C. The solvent was removed by vacuum and the residue was extracted with CH_2Cl_2/H_2O twice. The combined organic layers were washed with brine and dried with anhydrous $MgSO_4$. The solution was concentrated and further purified by thin-layer column chromatography with hexane/CH_2Cl_2 (1:1) as eluent. Product **1** was obtained as a light-yellow solid (12.6 mg, 50%). Single crystals of **1** suitable for X-ray diffraction were cultivated from its CH_2Cl_2 solution.

^{1}H NMR (400 MHz, $CDCl_3$): δ 8.31 (d, *J* = 7.3 Hz, 2H), 8.24 (d, *J* = 6.9 Hz, 2H), 7.81 (d, *J* = 7.9 Hz, 2H), 7.75 (d, *J* = 7.9 Hz, 2H), 7.52 (t, *J* = 7.7 Hz, 2H), 6.87 (t, *J* = 6.8 Hz, 2H).

^{13}C NMR (101 MHz, $CDCl_3$): δ 135.6, 134.4, 131.1, 129.6, 128.9, 127.7, 125.7, 120.9, 115.4.

^{11}B NMR (193 MHz, $CDCl_3$): δ 29.00.

HRMS (EI+, 70 eV): calculated for $C_{18}H_{12}{}^{10}BN$ $[M]^+$: 252.1099; found: 252.1102.

IV. Synthesis of *3a,9a*-diaza-*3a^1,6a^1*-dibora-perylene (2)

A mixture of 1,5-cyclooctadiene (COD, 540 mg, 5 mmol) and 2,2′-bipyridine (bpy, 390 mg, 2.5 mmol) in a Schlenk flask was dissolved in 30 mL of THF in the glovebox. $Ni(cod)_2$ (680 mg, 2.5 mmol) was added with magnetic stirring into the above colorless solution to afford a deep purple-blue solution, which was further stirred 5 min at room temperature. A solution of 4,5-dihalo-*4a,8a*-azaboranaphthalene (**3a**, 97 mg, 0.49 mmol; **3b**, 140 mg, 0.49 mmol) in 10 mL of THF was added into the above purple-blue solution. The sealed Schlenk flask was taken out of the glovebox and the reaction mixture was stirred for 12 hr at room temperature. The solvent was removed under vacuum and the residue was extracted with refluxing toluene (30 mL). After filtration and removal of toluene under vacuum, product **2** was isolated as a light-yellow solid (from **3a**: 39 mg, 63%; from **3b**: 62 mg, 100%). Single crystals of **2** suitable for X-ray diffraction were cultivated from its toluene solution.

^1H NMR (400 MHz, $CDCl_3$): δ 8.05 (d, J = 6.8 Hz, 4H), 7.59 (d, J = 6.8 Hz, 4H), 6.71 (t, J = 6.8 Hz, 4H).

^{13}C NMR (101 MHz, $CDCl_3$): δ 132.0, 127.9, 115.0.

^{11}B NMR (193 MHz, $CDCl_3$): δ 30.36.

HRMS (EI+, 70 eV): calculated for $C_{16}H_{12}{}^{10}B_2N_2$ $[M]^+$: 252.1259; found: 252.1252.

V. Determination of Structures of 1, 2, and 4

Single crystals of **1**, **2**, and **4** were immersed in FOMBLIN oil (HVAC 140/13, Sigma-Aldrich), mounted on a glass fiber, and examined on a Bruker D8 VENTURE diffractometer equipped with an Oxford Cryostream 800 low-temperature device using a nickel-filtered Cu Kα radiation source (λ= 1.54178 Å) and a Bruker PHOTON II detector at 150 K and 298 K, respectively. All data were integrated with SAINT and a multi-scan absorption correction using TWINABS was applied. The

structures were solved by direct methods using SHELXT and refined by full-matrix least-squares methods against F^2 by SHELXL-2018/1. All non-hydrogen atoms were refined with anisotropic displacement parameters. The hydrogen atoms were refined their pivot atoms for terminal sp^3 carbon atoms and 1.2 times for all other carbon atoms.

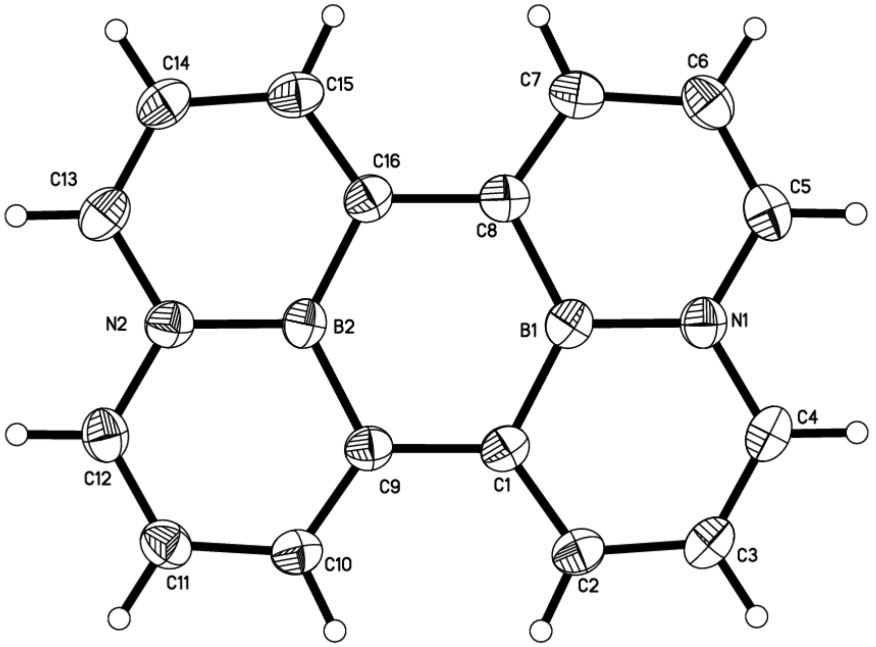

Table S1. Crystallographic data for 2

Identification code	191206b_a	
CCDC Deposition Number	2107103	
Empirical formula	C16 H12 B2 N2	
Formula weight	253.90	
Temperature	154(2) K	
Wavelength	1.54178 Å	
Crystal system	Monoclinic	
Space group	$P2_1/c$	
Unit cell dimensions	a = 8.025(2) Å	α= 90 °

	b = 11.722(3) Å	β = 100.484(19) °
	c = 13.149(4) Å	γ = 90 °
Volume	1216.3(6) $Å^3$	
Z	4	
Density (calculated)	1.386 Mg/m^3	
Absorption coefficient	0.617 mm^{-1}	
F(000)	528	
Crystal size	0.160 x 0.080 x 0.020 mm^3	
Theta range for data collection	5.093 to 66.593 °.	
Index ranges	-9<=h<=7, -10<=k<=13, -14<=l<=15	
Reflections collected	6537	
Independent reflections	2095 [R(int) = 0.0522]	
Completeness to theta = 66.593 °	97.8 %	
Absorption correction	Semi-empirical from equivalents	
Refinement method	Full-matrix least-squares on F^2	
Data / restraints / parameters	2095 / 0 / 181	
Goodness-of-fit on F^2	1.101	
Final R indices [I>2sigma(I)]	R1 = 0.0764, wR2 = 0.1811	
R indices (all data)	R1 = 0.1206, wR2 = 0.2023	
Largest diff. peak and hole	0.185 and -0.180 e.$Å^{-3}$	

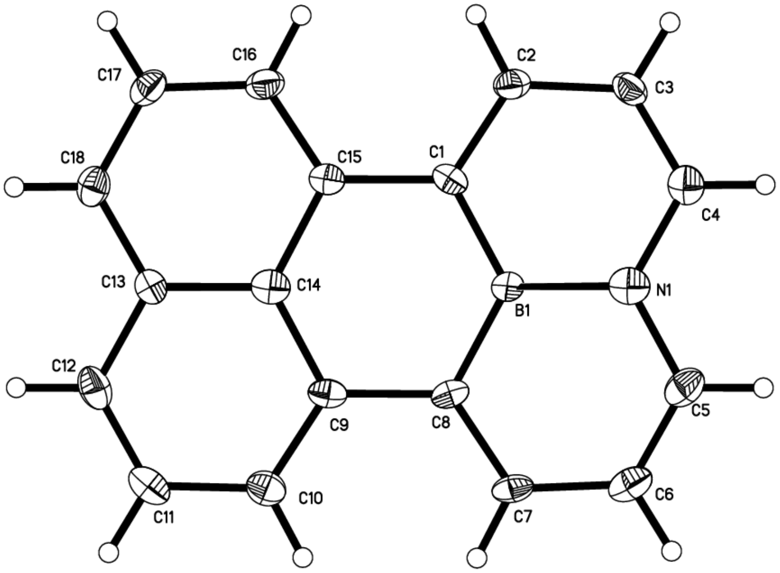

Table S2. Crystallographic data for 1

Identification code	191108c_a	
CCDC Deposition Number	2107101	
Empirical formula	C18 H12 B N	
Formula weight	253.10	
Temperature	150(2) K	
Wavelength	1.54178 Å	
Crystal system	Monoclinic	
Space group	$P2_1/c$	
Unit cell dimensions	a = 10.0992(3) Å	α = 90 °
	b = 10.8731(4) Å	β = 98.743(2) °
	c = 11.1388(3) Å	γ = 90 °
Volume	1208.93(7) $Å^3$	
Z	4	
Density (calculated)	1.391 Mg/m^3	
Absorption coefficient	0.609 mm^{-1}	
F(000)	528	
Crystal size	0.180 x 0.150 x 0.020 mm^3	
Theta range for data collection	4.429 to 66.535 °.	
Index ranges	-11<=h<=11, -12<=k<=8, -13<=l<=10	
Reflections collected	5175	
Independent reflections	2098 [R(int) = 0.0357]	
Completeness to theta = 66.536 °	98.2 %	
Absorption correction	Semi-empirical from equivalents	
Refinement method	Full-matrix least-squares on F^2	
Data / restraints / parameters	2098 / 0 / 181	
Goodness-of-fit on F^2	1.064	
Final R indices [I>2sigma(I)]	R1 = 0.0490, wR2 = 0.1392	
R indices (all data)	R1 = 0.0636, wR2 = 0.1507	
Largest diff. peak and hole	0.202 and -0.251 e.$Å^{-3}$	

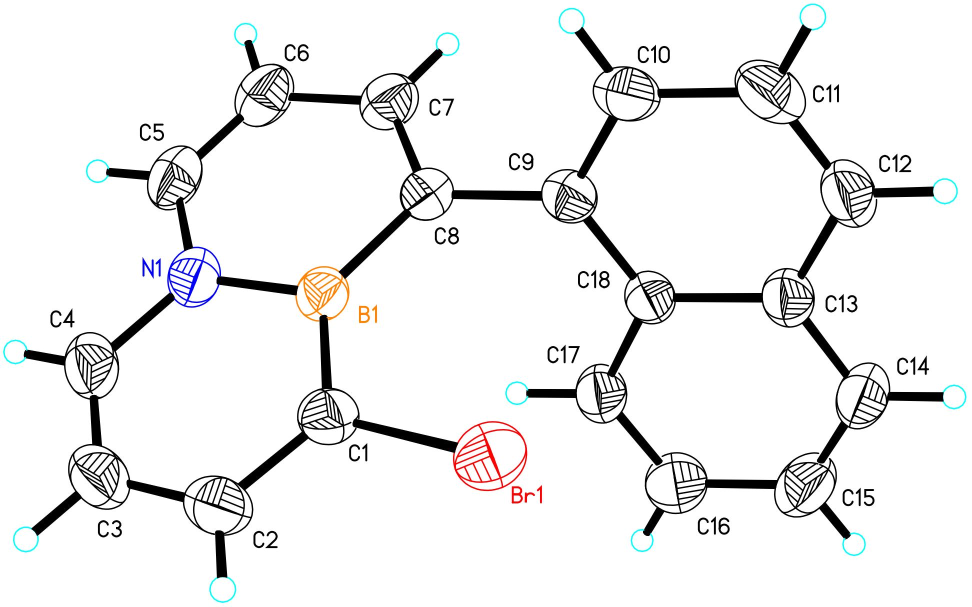

Table S3. **Crystallographic data for 4**

Identification code	191021c_a	
CCDC Deposition Number	2107102	
Empirical formula	C18 H13 B Br N	
Formula weight	334.01	
Temperature	298(2) K	
Wavelength	1.54178 Å	
Crystal system	Triclinic	
Space group	P-1	
Unit cell dimensions	a= 7.2364(4)Å	α= 108.647(2) °
	b= 9.8678(5)Å	β= 91.793(3) °
	c= 11.0736(6)Å	γ= 99.985(3) °
Volume	734.69(7) $Å^3$	
Z	2	
Density (calculated)	1.510 Mg/m^3	
Absorption coefficient	3.713 mm^{-1}	
F(000)	336	
Crystal size	0.250 x 0.130 x 0.050 mm^3	

Theta range for data collection	4.232 to 68.172 °.
Index ranges	-8<=h<=8, -11<=k<=11, -11<=l<=13
Reflections collected	6463
Independent reflections	2654 [R(int) = 0.0422]
Completeness to theta = 67.679 °	98.7 %
Absorption correction	Semi-empirical from equivalents
Refinement method	Full-matrix least-squares on F^2
Data / restraints / parameters	2654 / 0 / 190
Goodness-of-fit on F^2	1.047
Final R indices [I>2sigma(I)]	R1 = 0.0478, wR2 = 0.1286
R indices (all data)	R1 = 0.0517, wR2 = 0.1344
Largest diff. peak and hole	0.844 and -0.733 e.$Å^{-3}$

VI. Density Function Theory (DFT) Calculations

Density functional theory (DFT) calculations were carried out with Gaussian 09 software package.[1,2] The geometry optimization and frequency calculations were carried out at M062X/6-31G+(d) level.[3]

Perylene

File Name = Perylene

File Type = .log

Calculation Type = FOPT

Calculation Method = RM062X

Basis Set = 6-31+G(d)

Charge = 0

Spin = Singlet

E(RM062X) = -769.10449636 a.u.

RMS Gradient Norm = 0.00009880 a.u.

Imaginary Freq =

Dipole Moment = 0.0001 Debye

Point Group = C1

Job CPU time: 0 days 1 hours 40 minutes 54.9 seconds.

****

1.3500	0.2700	0.5934	C	0	0	0	0	0	0	0	0	0	0	0	0
2.6080	0.4642	1.1371	C	0	0	0	0	0	0	0	0	0	0	0	0
2.8968	1.5622	1.9710	C	0	0	0	0	0	0	0	0	0	0	0	0
1.9209	2.4785	2.2674	C	0	0	0	0	0	0	0	0	0	0	0	0
-0.3997	3.2710	2.0381	C	0	0	0	0	0	0	0	0	0	0	0	0
-1.6607	3.1173	1.5221	C	0	0	0	0	0	0	0	0	0	0	0	0
-1.9561	2.0209	0.6882	C	0	0	0	0	0	0	0	0	0	0	0	0
-1.0027	1.0726	0.3615	C	0	0	0	0	0	0	0	0	0	0	0	0
1.0426	-0.8818	-0.2833	C	0	0	0	0	0	0	0	0	0	0	0	0
1.9959	-1.8302	-0.6097	C	0	0	0	0	0	0	0	0	0	0	0	0
1.7005	-2.9266	-1.4436	C	0	0	0	0	0	0	0	0	0	0	0	0
0.4396	-3.0802	-1.9597	C	0	0	0	0	0	0	0	0	0	0	0	0
-1.8810	-2.2875	-2.1893	C	0	0	0	0	0	0	0	0	0	0	0	0
-2.8568	-1.3711	-1.8930	C	0	0	0	0	0	0	0	0	0	0	0	0
-2.5680	-0.2732	-1.0591	C	0	0	0	0	0	0	0	0	0	0	0	0
-1.3100	-0.0790	-0.5153	C	0	0	0	0	0	0	0	0	0	0	0	0
3.4093	-0.2358	0.9300	H	0	0	0	0	0	0	0	0	0	0	0	0
3.8984	1.6737	2.3754	H	0	0	0	0	0	0	0	0	0	0	0	0
2.1305	3.3303	2.9093	H	0	0	0	0	0	0	0	0	0	0	0	0
-0.1570	4.1127	2.6817	H	0	0	0	0	0	0	0	0	0	0	0	0
-2.4412	3.8374	1.7493	H	0	0	0	0	0	0	0	0	0	0	0	0
-2.9657	1.9387	0.3026	H	0	0	0	0	0	0	0	0	0	0	0	0
3.0055	-1.7480	-0.2240	H	0	0	0	0	0	0	0	0	0	0	0	0

2.4810	-3.6468	-1.6707	H	0	0	0	0	0	0	0	0	0	0	0	0
0.1969	-3.9220	-2.6033	H	0	0	0	0	0	0	0	0	0	0	0	0
-2.0906	-3.1394	-2.8312	H	0	0	0	0	0	0	0	0	0	0	0	0
-3.8584	-1.4826	-2.2975	H	0	0	0	0	0	0	0	0	0	0	0	0
-3.3693	0.4269	-0.8521	H	0	0	0	0	0	0	0	0	0	0	0	0
0.6146	2.3270	1.7364	C	0	0	0	0	0	0	0	0	0	0	0	0
0.3188	1.2161	0.8919	C	0	0	0	0	0	0	0	0	0	0	0	0
-0.2788	-1.0252	-0.8138	C	0	0	0	0	0	0	0	0	0	0	0	0
-0.5747	-2.1361	-1.6581	C	0	0	0	0	0	0	0	0	0	0	0	0

BN-Perylene

File Name = BN-Perylene

File Type = .log

Calculation Type = FOPT

Calculation Method = RM062X

Basis Set = 6-31+G(d)

Charge = 0

Spin = Singlet

E(RM062X) = -772.52114551 a.u.

RMS Gradient Norm = 0.00004385 a.u.

Imaginary Freq =

Dipole Moment = 1.7306 Debye

Point Group = C1

Job CPU time: 0 days 1 hours 36 minutes 35.3 seconds.

****

2.8961	0.0000	0.0000	N	0	0	0	0	0	0	0	0	0	0	0	0
0.7113	-1.3240	-0.0000	C	0	0	0	0	0	0	0	0	0	0	0	0
1.4779	-2.4641	-0.0001	C	0	0	0	0	0	0	0	0	0	0	0	0

2.9053	-2.3868	-0.0001	C	0	0	0	0	0	0	0	0	0	0	0	0
3.5748	-1.2002	-0.0000	C	0	0	0	0	0	0	0	0	0	0	0	0
3.5748	1.2002	0.0000	C	0	0	0	0	0	0	0	0	0	0	0	0
2.9053	2.3868	0.0001	C	0	0	0	0	0	0	0	0	0	0	0	0
1.4779	2.4641	0.0000	C	0	0	0	0	0	0	0	0	0	0	0	0
0.7113	1.3240	0.0000	C	0	0	0	0	0	0	0	0	0	0	0	0
-0.7656	-1.2749	0.0000	C	0	0	0	0	0	0	0	0	0	0	0	0
-1.5193	-2.4359	0.0001	C	0	0	0	0	0	0	0	0	0	0	0	0
-2.9270	-2.4223	0.0001	C	0	0	0	0	0	0	0	0	0	0	0	0
-3.5925	-1.2242	0.0000	C	0	0	0	0	0	0	0	0	0	0	0	0
-3.5925	1.2242	-0.0000	C	0	0	0	0	0	0	0	0	0	0	0	0
-2.9270	2.4223	-0.0001	C	0	0	0	0	0	0	0	0	0	0	0	0
-1.5193	2.4359	-0.0000	C	0	0	0	0	0	0	0	0	0	0	0	0
-0.7656	1.2749	-0.0000	C	0	0	0	0	0	0	0	0	0	0	0	0
1.4439	0.0000	0.0000	B	0	0	0	0	0	0	0	0	0	0	0	0
1.0427	-3.4622	-0.0001	H	0	0	0	0	0	0	0	0	0	0	0	0
3.4897	-3.3009	-0.0001	H	0	0	0	0	0	0	0	0	0	0	0	0
4.6595	-1.1419	-0.0000	H	0	0	0	0	0	0	0	0	0	0	0	0
4.6595	1.1419	0.0000	H	0	0	0	0	0	0	0	0	0	0	0	0
3.4897	3.3009	0.0001	H	0	0	0	0	0	0	0	0	0	0	0	0
1.0427	3.4622	0.0001	H	0	0	0	0	0	0	0	0	0	0	0	0
-1.0147	-3.3973	0.0001	H	0	0	0	0	0	0	0	0	0	0	0	0
-3.4749	-3.3601	0.0001	H	0	0	0	0	0	0	0	0	0	0	0	0
-4.6792	-1.1893	0.0000	H	0	0	0	0	0	0	0	0	0	0	0	0
-4.6792	1.1893	-0.0000	H	0	0	0	0	0	0	0	0	0	0	0	0
-3.4749	3.3601	-0.0001	H	0	0	0	0	0	0	0	0	0	0	0	0
-1.0147	3.3973	-0.0001	H	0	0	0	0	0	0	0	0	0	0	0	0
-1.4453	0.0000	-0.0000	C	0	0	0	0	0	0	0	0	0	0	0	0

-2.8741 -0.0000 -0.0000 C 0 0 0 0 0 0 0 0 0 0 0 0

2BN-Perylene

File Name = 2BN-Perylene

File Type = .log

Calculation Type = FOPT

Calculation Method = RM062X

Basis Set = 6-31+G(d)

Charge = 0

Spin = Singlet

E(RM062X) = -775.94109280 a.u.

RMS Gradient Norm = 0.00008262 a.u.

Imaginary Freq =

Dipole Moment = 0.0000 Debye

Point Group = C1

Job CPU time: 0 days 1 hours 11 minutes 2.5 seconds.

****

0.6224 2.3565 1.7604 N 0 0 0 0 0 0 0 0 0 0 0 0

-0.5824 -2.1657 -1.6821 N 0 0 0 0 0 0 0 0 0 0 0 0

1.4533 0.2353 0.6027 C 0 0 0 0 0 0 0 0 0 0 0 0

2.6766 0.4870 1.1713 C 0 0 0 0 0 0 0 0 0 0 0 0

2.8858 1.6259 2.0101 C 0 0 0 0 0 0 0 0 0 0 0 0

1.8913 2.5128 2.2839 C 0 0 0 0 0 0 0 0 0 0 0 0

-0.3582 3.2827 2.0590 C 0 0 0 0 0 0 0 0 0 0 0 0

-1.6200 3.1647 1.5647 C 0 0 0 0 0 0 0 0 0 0 0 0

-2.0032 2.0820 0.7125 C 0 0 0 0 0 0 0 0 0 0 0 0

-1.1068 1.1075 0.3518 C 0 0 0 0 0 0 0 0 0 0 0 0

1.1467 -0.9166 -0.2735 C 0 0 0 0 0 0 0 0 0 0 0 0

2.0431	-1.8912	-0.6342	C	0	0	0	0	0	0	0	0	0	0	0	0
1.6598	-2.9739	-1.4864	C	0	0	0	0	0	0	0	0	0	0	0	0
0.3981	-3.0918	-1.9807	C	0	0	0	0	0	0	0	0	0	0	0	0
-1.8513	-2.3219	-2.2057	C	0	0	0	0	0	0	0	0	0	0	0	0
-2.8458	-1.4349	-1.9319	C	0	0	0	0	0	0	0	0	0	0	0	0
-2.6366	-0.2961	-1.0932	C	0	0	0	0	0	0	0	0	0	0	0	0
-1.4133	-0.0444	-0.5244	C	0	0	0	0	0	0	0	0	0	0	0	0
0.3199	1.2223	0.8971	B	0	0	0	0	0	0	0	0	0	0	0	0
-0.2800	-1.0314	-0.8188	B	0	0	0	0	0	0	0	0	0	0	0	0
3.5307	-0.1693	1.0060	H	0	0	0	0	0	0	0	0	0	0	0	0
3.8628	1.7995	2.4490	H	0	0	0	0	0	0	0	0	0	0	0	0
2.0433	3.3800	2.9205	H	0	0	0	0	0	0	0	0	0	0	0	0
-0.0608	4.1033	2.7058	H	0	0	0	0	0	0	0	0	0	0	0	0
-2.3482	3.9230	1.8334	H	0	0	0	0	0	0	0	0	0	0	0	0
-3.0358	2.0687	0.3645	H	0	0	0	0	0	0	0	0	0	0	0	0
3.0757	-1.8779	-0.2861	H	0	0	0	0	0	0	0	0	0	0	0	0
2.3880	-3.7322	-1.7550	H	0	0	0	0	0	0	0	0	0	0	0	0
0.1007	-3.9124	-2.6275	H	0	0	0	0	0	0	0	0	0	0	0	0
-2.0034	-3.1891	-2.8423	H	0	0	0	0	0	0	0	0	0	0	0	0
-3.8228	-1.6085	-2.3709	H	0	0	0	0	0	0	0	0	0	0	0	0
-3.4907	0.3603	-0.9279	H	0	0	0	0	0	0	0	0	0	0	0	0

Perylene-D

File Name = Perylene-D

File Type = .log

Calculation Type = FOPT

Calculation Method = RM062X

Basis Set = 6-31+G(d)

Charge = 0

Spin = Singlet

E(RM062X) = -1538.23696969 a.u.

RMS Gradient Norm = 0.00003060 a.u.

Imaginary Freq =

Dipole Moment = 0.0010 Debye

Point Group = C1

Job CPU time: 7 days 6 hours 20 minutes 19.1 seconds.

****

0.9323	-0.0062	0.4442	C	0	0	0	0	0	0	0	0	0	0	0	0
2.1396	0.0523	1.1183	C	0	0	0	0	0	0	0	0	0	0	0	0
2.4289	1.0831	2.0335	C	0	0	0	0	0	0	0	0	0	0	0	0
1.5043	2.0632	2.2868	C	0	0	0	0	0	0	0	0	0	0	0	0
-0.7214	3.0426	1.9012	C	0	0	0	0	0	0	0	0	0	0	0	0
-1.9392	3.0107	1.2707	C	0	0	0	0	0	0	0	0	0	0	0	0
-2.2311	1.9904	0.3442	C	0	0	0	0	0	0	0	0	0	0	0	0
-1.3184	0.9949	0.0409	C	0	0	0	0	0	0	0	0	0	0	0	0
0.6241	-1.0831	-0.5220	C	0	0	0	0	0	0	0	0	0	0	0	0
1.5241	-2.0963	-0.8032	C	0	0	0	0	0	0	0	0	0	0	0	0
1.2336	-3.1172	-1.7290	C	0	0	0	0	0	0	0	0	0	0	0	0
0.0304	-3.1280	-2.3861	C	0	0	0	0	0	0	0	0	0	0	0	0
-2.1813	-2.1297	-2.7987	C	0	0	0	0	0	0	0	0	0	0	0	0
-3.1186	-1.1668	-2.5268	C	0	0	0	0	0	0	0	0	0	0	0	0
-2.8394	-0.1521	-1.5893	C	0	0	0	0	0	0	0	0	0	0	0	0
-1.6256	-0.0826	-0.9260	C	0	0	0	0	0	0	0	0	0	0	0	0
2.8927	-0.7126	0.9656	H	0	0	0	0	0	0	0	0	0	0	0	0
3.3881	1.0850	2.5440	H	0	0	0	0	0	0	0	0	0	0	0	0
1.7145	2.8620	2.9941	H	0	0	0	0	0	0	0	0	0	0	0	0

-0.4816	3.8262	2.6161	H	0	0	0	0	0	0	0	0	0	0	0	0
-2.6858	3.7728	1.4758	H	0	0	0	0	0	0	0	0	0	0	0	0
-3.2048	2.0063	-0.1325	H	0	0	0	0	0	0	0	0	0	0	0	0
2.4812	-2.1353	-0.2941	H	0	0	0	0	0	0	0	0	0	0	0	0
1.9625	-3.9041	-1.9002	H	0	0	0	0	0	0	0	0	0	0	0	0
-0.2133	-3.9174	-3.0934	H	0	0	0	0	0	0	0	0	0	0	0	0
-2.3847	-2.9233	-3.5136	H	0	0	0	0	0	0	0	0	0	0	0	0
-4.0816	-1.1763	-3.0298	H	0	0	0	0	0	0	0	0	0	0	0	0
-3.6074	0.5918	-1.4077	H	0	0	0	0	0	0	0	0	0	0	0	0
0.6698	-3.1986	2.3694	C	0	0	0	0	0	0	0	0	0	0	0	0
1.8836	-3.1291	3.0330	C	0	0	0	0	0	0	0	0	0	0	0	0
2.1625	-2.1145	3.9706	C	0	0	0	0	0	0	0	0	0	0	0	0
1.2252	-1.1515	4.2423	C	0	0	0	0	0	0	0	0	0	0	0	0
-0.9865	-0.1533	3.8295	C	0	0	0	0	0	0	0	0	0	0	0	0
-2.1896	-0.1640	3.1721	C	0	0	0	0	0	0	0	0	0	0	0	0
-2.4798	-1.1848	2.2460	C	0	0	0	0	0	0	0	0	0	0	0	0
-1.5799	-2.1982	1.9652	C	0	0	0	0	0	0	0	0	0	0	0	0
0.3630	-4.2760	1.4024	C	0	0	0	0	0	0	0	0	0	0	0	0
1.2758	-5.2713	1.0990	C	0	0	0	0	0	0	0	0	0	0	0	0
0.9843	-6.2913	0.1721	C	0	0	0	0	0	0	0	0	0	0	0	0
-0.2336	-6.3234	-0.4584	C	0	0	0	0	0	0	0	0	0	0	0	0
-2.4597	-5.3448	-0.8434	C	0	0	0	0	0	0	0	0	0	0	0	0
-3.3846	-4.3651	-0.5897	C	0	0	0	0	0	0	0	0	0	0	0	0
-3.0955	-3.3343	0.3255	C	0	0	0	0	0	0	0	0	0	0	0	0
-1.8880	-3.2753	0.9992	C	0	0	0	0	0	0	0	0	0	0	0	0
2.6516	-3.8730	2.8514	H	0	0	0	0	0	0	0	0	0	0	0	0
3.1255	-2.1049	4.4736	H	0	0	0	0	0	0	0	0	0	0	0	0
1.4285	-0.3580	4.9572	H	0	0	0	0	0	0	0	0	0	0	0	0

-0.7431	0.6356	4.5374	H	0	0	0	0	0	0	0	0	0	0	0	0
-2.9185	0.6228	3.3434	H	0	0	0	0	0	0	0	0	0	0	0	0
-3.4366	-1.1456	1.7366	H	0	0	0	0	0	0	0	0	0	0	0	0
2.2495	-5.2871	1.5759	H	0	0	0	0	0	0	0	0	0	0	0	0
1.7311	-7.0531	-0.0332	H	0	0	0	0	0	0	0	0	0	0	0	0
-0.4731	-7.1069	-1.1735	H	0	0	0	0	0	0	0	0	0	0	0	0
-2.6697	-6.1436	-1.5508	H	0	0	0	0	0	0	0	0	0	0	0	0
-4.3439	-4.3674	-1.1000	H	0	0	0	0	0	0	0	0	0	0	0	0
-3.8489	-2.5698	0.4786	H	0	0	0	0	0	0	0	0	0	0	0	0
0.2482	2.0451	1.6288	C	0	0	0	0	0	0	0	0	0	0	0	0
-0.0465	1.0071	0.6957	C	0	0	0	0	0	0	0	0	0	0	0	0
-0.0256	-1.1643	3.5758	C	0	0	0	0	0	0	0	0	0	0	0	0
-0.3157	-2.1970	2.6352	C	0	0	0	0	0	0	0	0	0	0	0	0
-0.6401	-1.0843	-1.1918	C	0	0	0	0	0	0	0	0	0	0	0	0
-0.9304	-2.1170	-2.1324	C	0	0	0	0	0	0	0	0	0	0	0	0
-0.9090	-4.2883	0.7475	C	0	0	0	0	0	0	0	0	0	0	0	0
-1.2035	-5.3264	-0.1856	C	0	0	0	0	0	0	0	0	0	0	0	0

2BN-Perylene-D

File Name = 2BN-Perylene-D

File Type = .log

Calculation Type = FOPT

Calculation Method = RM062X

Basis Set = 6-31+G(d)

Charge = 0

Spin = Singlet

E(RM062X) = -1551.91154942 a.u.

RMS Gradient Norm = 0.00001444 a.u.

Imaginary Freq =

Dipole Moment = 0.0004 Debye

Point Group = C1

Job CPU time: 4 days 14 hours 22 minutes 47.6 seconds.

****

0.1712	2.1131	1.6164	N	0	0	0	0	0	0	0	0	0	0	0	0
-1.0531	-2.1050	-2.1870	N	0	0	0	0	0	0	0	0	0	0	0	0
0.9205	-0.0341	0.4503	C	0	0	0	0	0	0	0	0	0	0	0	0
2.0733	0.0443	1.1911	C	0	0	0	0	0	0	0	0	0	0	0	0
2.2886	1.1165	2.1128	C	0	0	0	0	0	0	0	0	0	0	0	0
1.3677	2.0994	2.3070	C	0	0	0	0	0	0	0	0	0	0	0	0
-0.7542	3.1036	1.8762	C	0	0	0	0	0	0	0	0	0	0	0	0
-1.9598	3.1328	1.2456	C	0	0	0	0	0	0	0	0	0	0	0	0
-2.3362	2.1461	0.2804	C	0	0	0	0	0	0	0	0	0	0	0	0
-1.4919	1.1166	-0.0515	C	0	0	0	0	0	0	0	0	0	0	0	0
0.6078	-1.1077	-0.5181	C	0	0	0	0	0	0	0	0	0	0	0	0
1.4296	-2.1666	-0.8087	C	0	0	0	0	0	0	0	0	0	0	0	0
1.0429	-3.1712	-1.7509	C	0	0	0	0	0	0	0	0	0	0	0	0
-0.1503	-3.1281	-2.4019	C	0	0	0	0	0	0	0	0	0	0	0	0
-2.2608	-2.1102	-2.8520	C	0	0	0	0	0	0	0	0	0	0	0	0
-3.1954	-1.1439	-2.6392	C	0	0	0	0	0	0	0	0	0	0	0	0
-2.9767	-0.0688	-1.7215	C	0	0	0	0	0	0	0	0	0	0	0	0
-1.8049	0.0383	-1.0148	C	0	0	0	0	0	0	0	0	0	0	0	0
-0.1334	1.0587	0.6578	B	0	0	0	0	0	0	0	0	0	0	0	0
-0.7466	-1.0483	-1.2300	B	0	0	0	0	0	0	0	0	0	0	0	0
2.8548	-0.7121	1.1173	H	0	0	0	0	0	0	0	0	0	0	0	0
3.2082	1.1536	2.6883	H	0	0	0	0	0	0	0	0	0	0	0	0
1.5263	2.9145	3.0082	H	0	0	0	0	0	0	0	0	0	0	0	0

-0.4624	3.8528	2.6075	H	0	0	0	0	0	0	0	0	0	0	0	0
-2.6450	3.9383	1.4899	H	0	0	0	0	0	0	0	0	0	0	0	0
-3.3232	2.2469	-0.1710	H	0	0	0	0	0	0	0	0	0	0	0	0
2.3953	-2.2941	-0.3193	H	0	0	0	0	0	0	0	0	0	0	0	0
1.7010	-4.0133	-1.9410	H	0	0	0	0	0	0	0	0	0	0	0	0
-0.4581	-3.8984	-3.1046	H	0	0	0	0	0	0	0	0	0	0	0	0
-2.4235	-2.9319	-3.5440	H	0	0	0	0	0	0	0	0	0	0	0	0
-4.1275	-1.2010	-3.1922	H	0	0	0	0	0	0	0	0	0	0	0	0
-3.7776	0.6634	-1.6153	H	0	0	0	0	0	0	0	0	0	0	0	0
0.0973	-1.1767	3.6309	N	0	0	0	0	0	0	0	0	0	0	0	0
-1.1271	-5.3945	-0.1729	N	0	0	0	0	0	0	0	0	0	0	0	0
0.8495	-3.3193	2.4576	C	0	0	0	0	0	0	0	0	0	0	0	0
2.0218	-3.2115	3.1634	C	0	0	0	0	0	0	0	0	0	0	0	0
2.2405	-2.1364	4.0812	C	0	0	0	0	0	0	0	0	0	0	0	0
1.3052	-1.1711	4.2953	C	0	0	0	0	0	0	0	0	0	0	0	0
-0.8055	-0.1535	3.8457	C	0	0	0	0	0	0	0	0	0	0	0	0
-1.9985	-0.1103	3.1945	C	0	0	0	0	0	0	0	0	0	0	0	0
-2.3853	-1.1148	2.2522	C	0	0	0	0	0	0	0	0	0	0	0	0
-1.5636	-2.1738	1.9617	C	0	0	0	0	0	0	0	0	0	0	0	0
0.5364	-4.3977	1.4946	C	0	0	0	0	0	0	0	0	0	0	0	0
1.3806	-5.4274	1.1628	C	0	0	0	0	0	0	0	0	0	0	0	0
1.0040	-6.4142	0.1978	C	0	0	0	0	0	0	0	0	0	0	0	0
-0.2017	-6.3850	-0.4327	C	0	0	0	0	0	0	0	0	0	0	0	0
-2.3238	-5.3811	-0.8631	C	0	0	0	0	0	0	0	0	0	0	0	0
-3.2448	-4.3983	-0.6687	C	0	0	0	0	0	0	0	0	0	0	0	0
-3.0294	-3.3261	0.2531	C	0	0	0	0	0	0	0	0	0	0	0	0
-1.8762	-3.2472	0.9932	C	0	0	0	0	0	0	0	0	0	0	0	0
-0.2091	-2.2332	2.6736	B	0	0	0	0	0	0	0	0	0	0	0	0

-0.8223	-4.3399	0.7854	B	0	0	0	0	0	0	0	0	0	0	0	0
2.8232	-3.9431	3.0564	H	0	0	0	0	0	0	0	0	0	0	0	0
3.1730	-2.0788	4.6334	H	0	0	0	0	0	0	0	0	0	0	0	0
1.4680	-0.3494	4.9873	H	0	0	0	0	0	0	0	0	0	0	0	0
-0.4976	0.6168	4.5484	H	0	0	0	0	0	0	0	0	0	0	0	0
-2.6566	0.7320	3.3844	H	0	0	0	0	0	0	0	0	0	0	0	0
-3.3508	-0.9869	1.7625	H	0	0	0	0	0	0	0	0	0	0	0	0
2.3674	-5.5284	1.6145	H	0	0	0	0	0	0	0	0	0	0	0	0
1.6891	-7.2197	-0.0465	H	0	0	0	0	0	0	0	0	0	0	0	0
-0.4935	-7.1343	-1.1639	H	0	0	0	0	0	0	0	0	0	0	0	0
-2.4825	-6.1962	-1.5642	H	0	0	0	0	0	0	0	0	0	0	0	0
-4.1646	-4.4357	-1.2438	H	0	0	0	0	0	0	0	0	0	0	0	0
-3.8113	-2.5701	0.3276	H	0	0	0	0	0	0	0	0	0	0	0	0

References:

[1] a) A. D. Becke, *J. Chem. Phys.* **1993**, *98*, 5648; b) P. J. Stephens, F. J. Devlin, C. F. Chabalowski, M. J. Frisch, *J. Phys. Chem.* **1994**, 98, 11623; c) C. Lee, W. Yang, R. G. Parr, *Phys. Rev. B: Condens. Matter Mater. Phys.* **1988**, *37*, 785.

[2] M. J. Frisch, G. W. Trucks, H. B. Schlegel, G. E. Scuseria, M. A. Robb, J. R. Cheeseman, G. Scalmani, V. Barone, B. Mennucci, G. A. Petersson, H. Nakatsuji, M. Caricato, X. Li, H. P. Hratchian, A. F. Izmaylov, J. Bloino, G. Zheng, J. L. Sonnenberg, M. Hada, M. Ehara, K. Toyota, R. Fukuda, J. Hasegawa, M. Ishida, T. Nakajima, Y. Honda, O. Kitao, H. Nakai, T. Vreven, J. A., Jr. Montgomery, J. E. Peralta, F. Ogliaro, M. Bearpark, J. J. Heyd, E. Brothers, K. N. Kudin, V. N. Staroverov, R. Kobayashi, J. Normand, K. Raghavachari, A. Rendell, J. C. Burant, S. S. Iyengar, J. Tomasi, M. Cossi, N. Rega, N. J. Millam, M. Klene, J. E. Knox, J. B. Cross, V. Bakken, C. Adamo, J. Jaramillo, R. Gomperts, R. E. Stratmann, O. Yazyev, A. J. Austin, R. Cammi, C. Pomelli, J. W. Ochterski, R. L. Martin, K. Morokuma, V. G. Zakrzewski, G. A. Voth, P. Salvador, J. J. Dannenberg, S. Dapprich, A. D. Daniels, O. Farkas,

J. B. Foresman, J. V. Ortiz, J. Cioslowski, D. J. Fox, *Gaussian 09*, Revision D.01; Gaussian, Inc.: Wallingford, CT, 2009.

[3] Y. Zhao and D. G. Truhlar, *Theor. Chem. Acc.*, **2008**, *120*, 215.

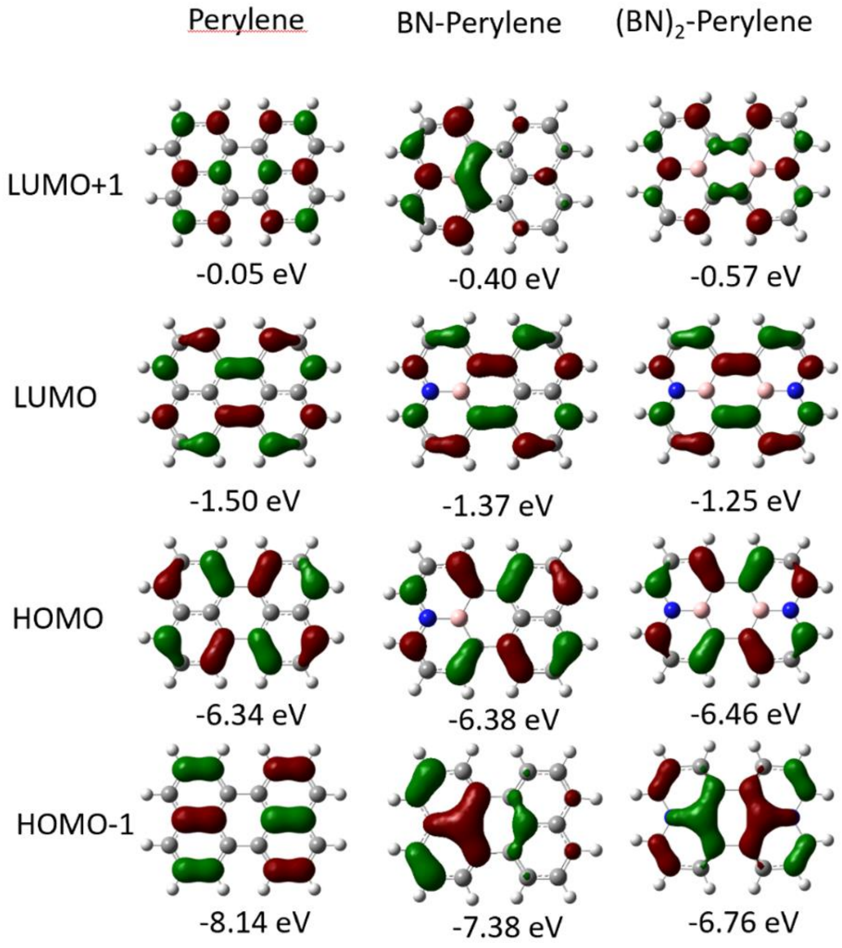

NMR Spectra and HRMS

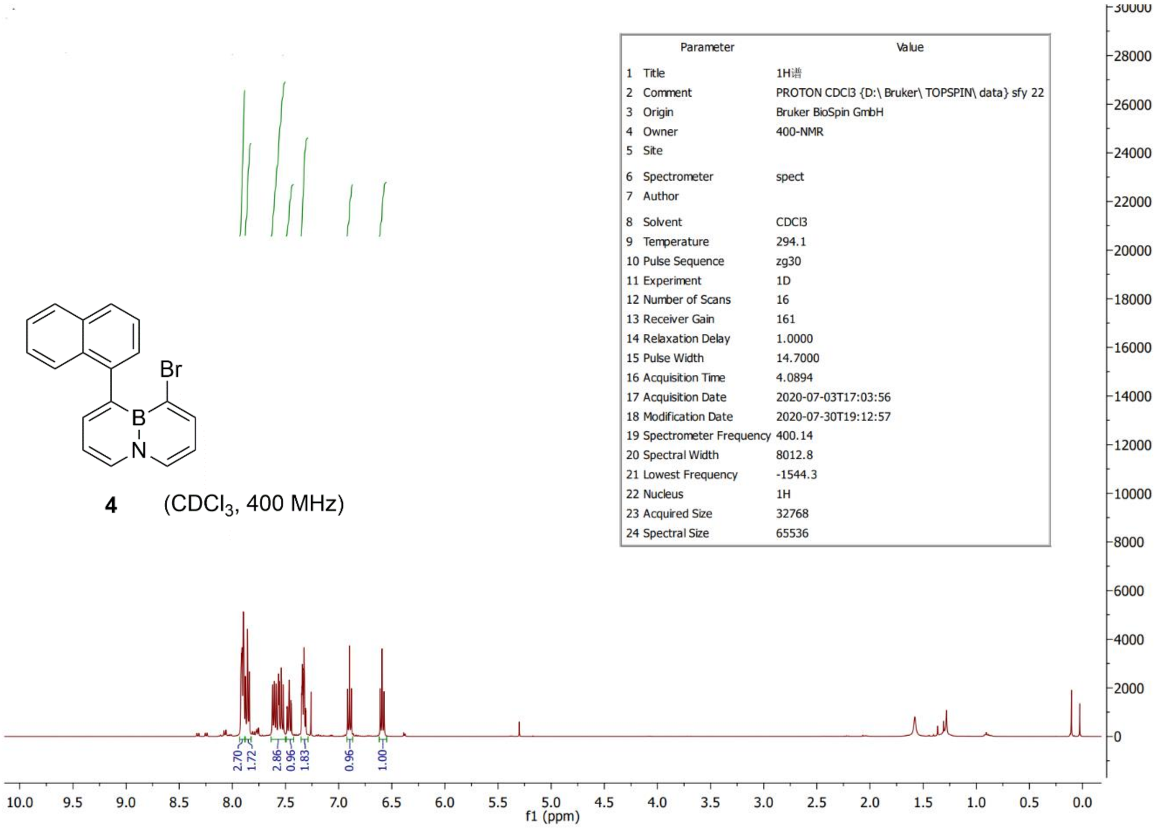

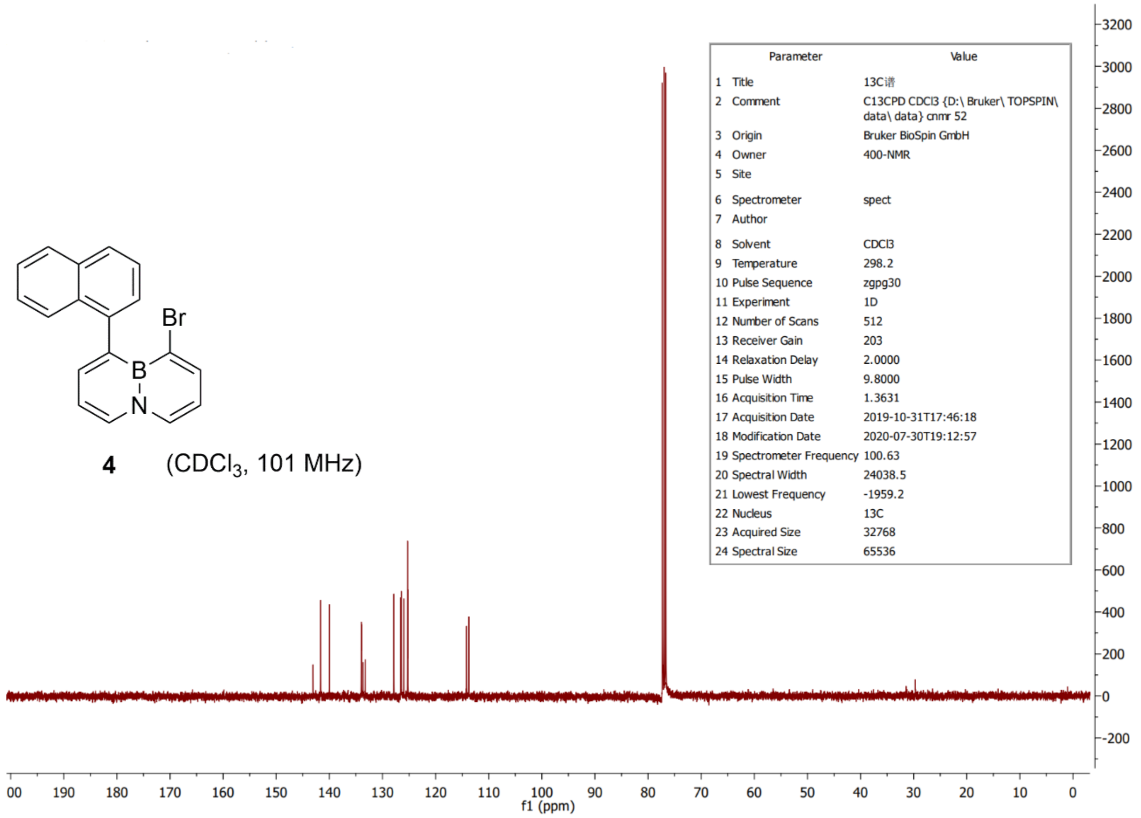

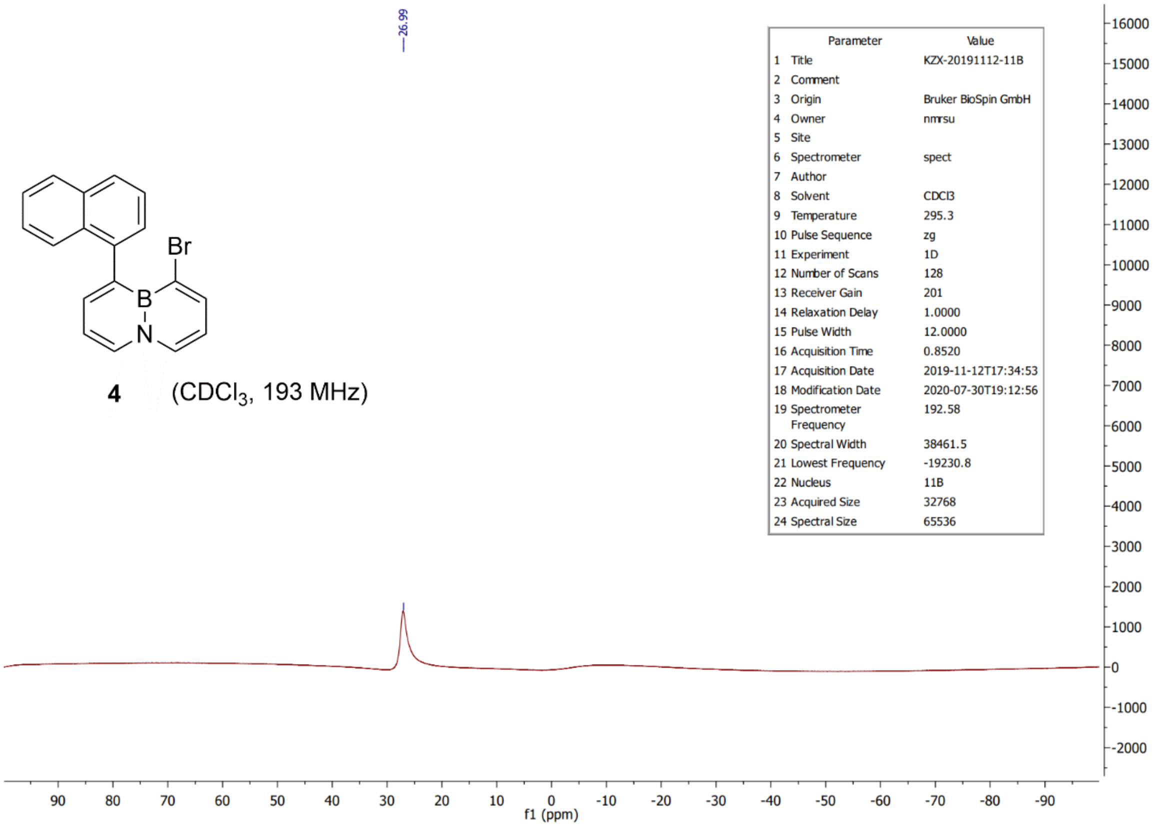

4 (CDCl$_3$, 193 MHz)

Parameter		Value
1	Title	KZX-20191112-11B
2	Comment	
3	Origin	Bruker BioSpin GmbH
4	Owner	nmrsu
5	Site	
6	Spectrometer	spect
7	Author	
8	Solvent	CDCl3
9	Temperature	295.3
10	Pulse Sequence	zg
11	Experiment	1D
12	Number of Scans	128
13	Receiver Gain	201
14	Relaxation Delay	1.0000
15	Pulse Width	12.0000
16	Acquisition Time	0.8520
17	Acquisition Date	2019-11-12T17:34:53
18	Modification Date	2020-07-30T19:12:56
19	Spectrometer Frequency	192.58
20	Spectral Width	38461.5
21	Lowest Frequency	-19230.8
22	Nucleus	11B
23	Acquired Size	32768
24	Spectral Size	65536

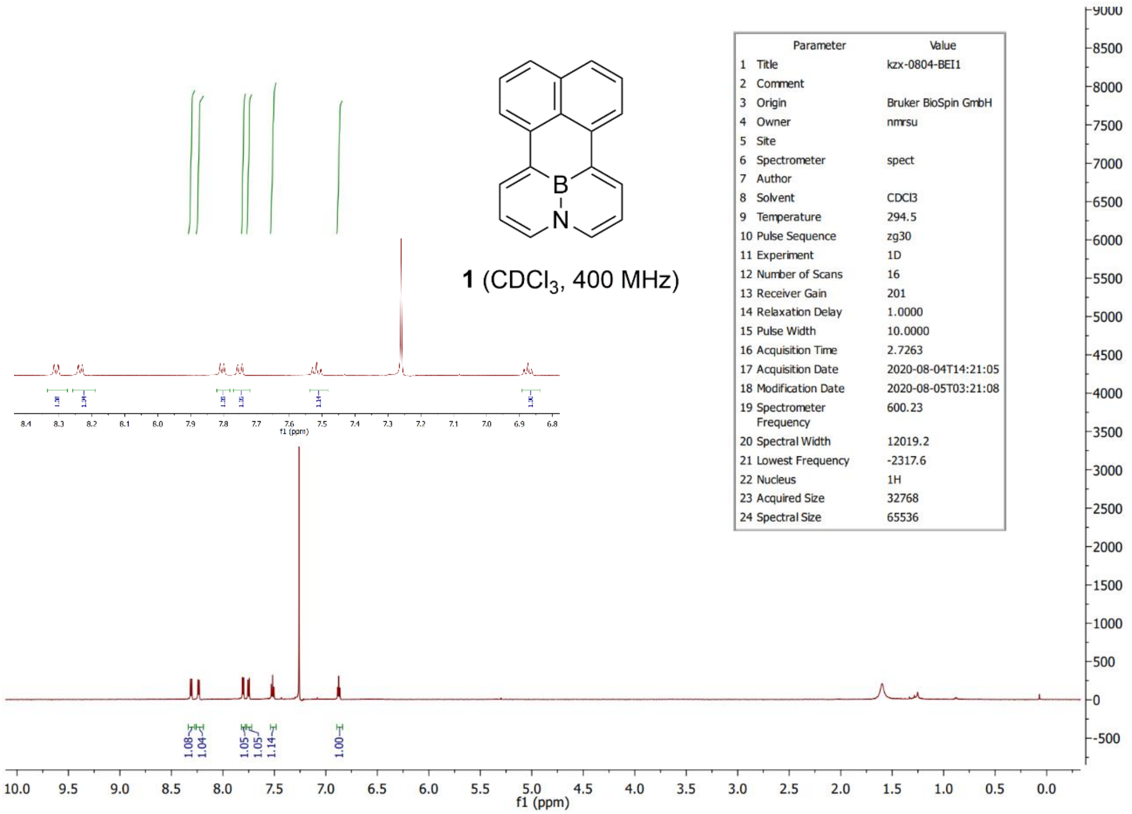

1 (CDCl$_3$, 400 MHz)

Parameter		Value
1	Title	kzx-0804-BEI1
2	Comment	
3	Origin	Bruker BioSpin GmbH
4	Owner	nmrsu
5	Site	
6	Spectrometer	spect
7	Author	
8	Solvent	CDCl3
9	Temperature	294.5
10	Pulse Sequence	zg30
11	Experiment	1D
12	Number of Scans	16
13	Receiver Gain	201
14	Relaxation Delay	1.0000
15	Pulse Width	10.0000
16	Acquisition Time	2.7263
17	Acquisition Date	2020-08-04T14:21:05
18	Modification Date	2020-08-05T03:21:08
19	Spectrometer Frequency	600.23
20	Spectral Width	12019.2
21	Lowest Frequency	-2317.6
22	Nucleus	1H
23	Acquired Size	32768
24	Spectral Size	65536

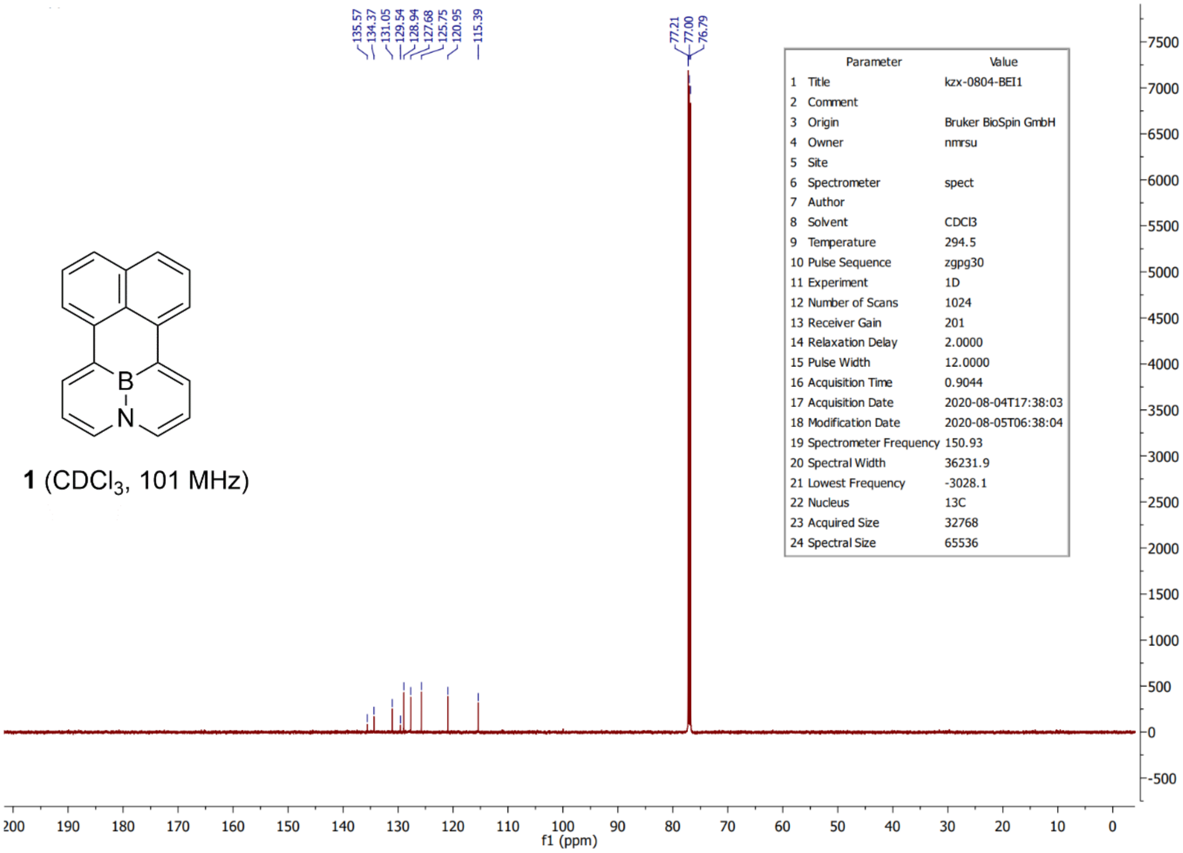
135.57
134.37
131.05
129.54
128.94
127.68
125.75
120.95
115.39
77.21
77.00
76.79
1 (CDCl3, 101 MHz)
Parameter Value
1 Title kzx-0804-BEI1
2 Comment
3 Origin Bruker BioSpin GmbH
4 Owner nmrsu
5 Site
6 Spectrometer spect
7 Author
8 Solvent CDCl3
9 Temperature 294.5
10 Pulse Sequence zgpg30
11 Experiment 1D
12 Number of Scans 1024
13 Receiver Gain 201
14 Relaxation Delay 2.0000
15 Pulse Width 12.0000
16 Acquisition Time 0.9044
17 Acquisition Date 2020-08-04T17:38:03
18 Modification Date 2020-08-05T06:38:04
19 Spectrometer Frequency 150.93
20 Spectral Width 36231.9
21 Lowest Frequency -3028.1
22 Nucleus 13C
23 Acquired Size 32768
24 Spectral Size 65536
f1 (ppm)

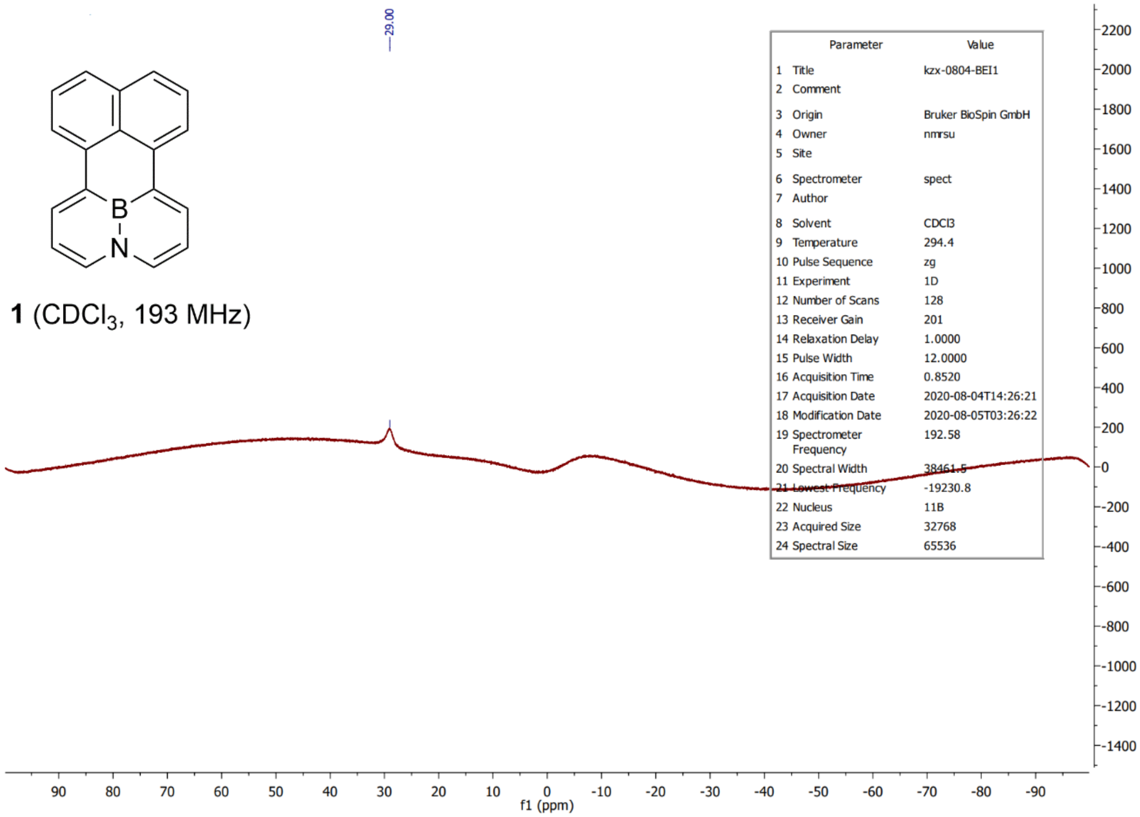
29.00
1 (CDCl3, 193 MHz)
Parameter Value
1 Title kzx-0804-BEI1
2 Comment
3 Origin Bruker BioSpin GmbH
4 Owner nmrsu
5 Site
6 Spectrometer spect
7 Author
8 Solvent CDCl3
9 Temperature 294.4
10 Pulse Sequence zg
11 Experiment 1D
12 Number of Scans 128
13 Receiver Gain 201
14 Relaxation Delay 1.0000
15 Pulse Width 12.0000
16 Acquisition Time 0.8520
17 Acquisition Date 2020-08-04T14:26:21
18 Modification Date 2020-08-05T03:26:22
19 Spectrometer Frequency 192.58
20 Spectral Width 38461.5
21 Lowest Frequency -19230.8
22 Nucleus 11B
23 Acquired Size 32768
24 Spectral Size 65536
f1 (ppm)

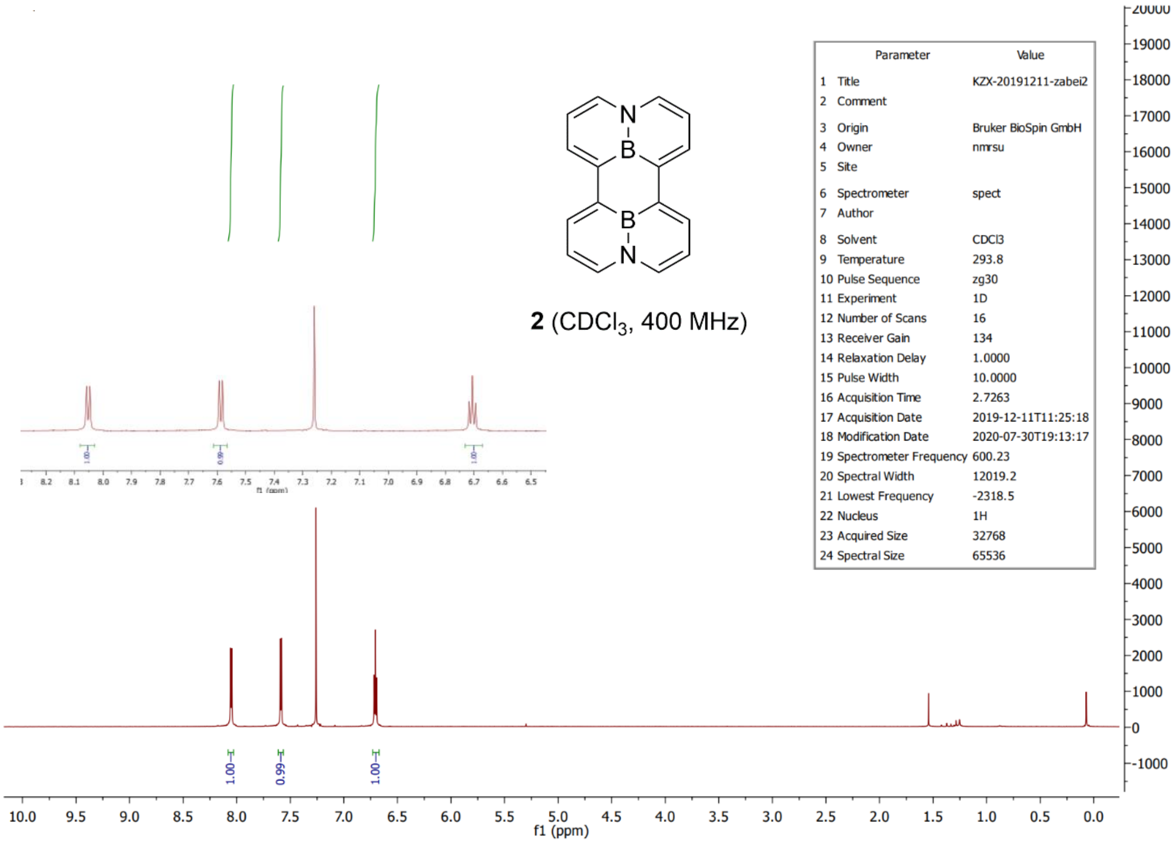
2 (CDCl3, 400 MHz)
Parameter Value
1 Title KZX-20191211-zabei2
2 Comment
3 Origin Bruker BioSpin GmbH
4 Owner nmrsu
5 Site
6 Spectrometer spect
7 Author
8 Solvent CDCl3
9 Temperature 293.8
10 Pulse Sequence zg30
11 Experiment 1D
12 Number of Scans 16
13 Receiver Gain 134
14 Relaxation Delay 1.0000
15 Pulse Width 10.0000
16 Acquisition Time 2.7263
17 Acquisition Date 2019-12-11T11:25:18
18 Modification Date 2020-07-30T19:13:17
19 Spectrometer Frequency 600.23
20 Spectral Width 12019.2
21 Lowest Frequency -2318.5
22 Nucleus 1H
23 Acquired Size 32768
24 Spectral Size 65536
1.00
0.99
1.00
f1 (ppm)

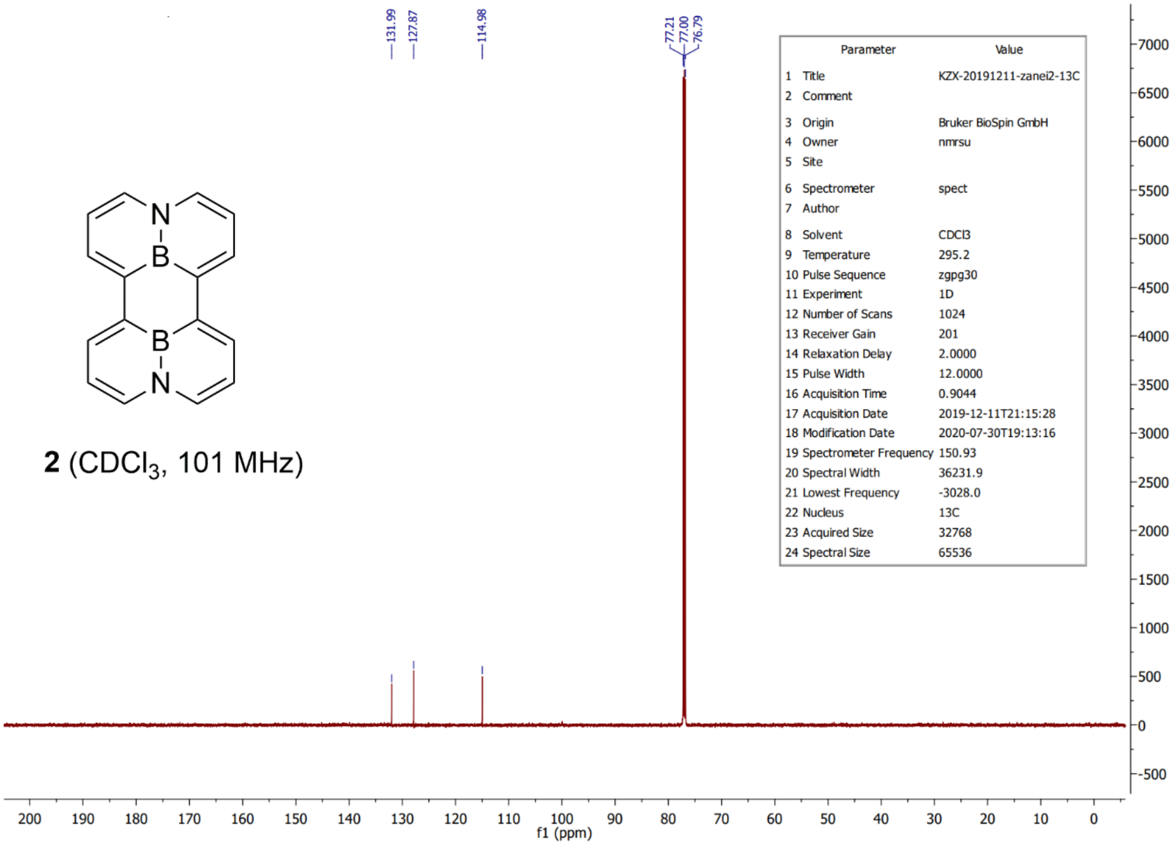
131.99
127.87
114.98
77.21
77.00
76.79
2 (CDCl3, 101 MHz)
Parameter Value
1 Title KZX-20191211-zanei2-13C
2 Comment
3 Origin Bruker BioSpin GmbH
4 Owner nmrsu
5 Site
6 Spectrometer spect
7 Author
8 Solvent CDCl3
9 Temperature 295.2
10 Pulse Sequence zgpg30
11 Experiment 1D
12 Number of Scans 1024
13 Receiver Gain 201
14 Relaxation Delay 2.0000
15 Pulse Width 12.0000
16 Acquisition Time 0.9044
17 Acquisition Date 2019-12-11T21:15:28
18 Modification Date 2020-07-30T19:13:16
19 Spectrometer Frequency 150.93
20 Spectral Width 36231.9
21 Lowest Frequency -3028.0
22 Nucleus 13C
23 Acquired Size 32768
24 Spectral Size 65536
f1 (ppm)

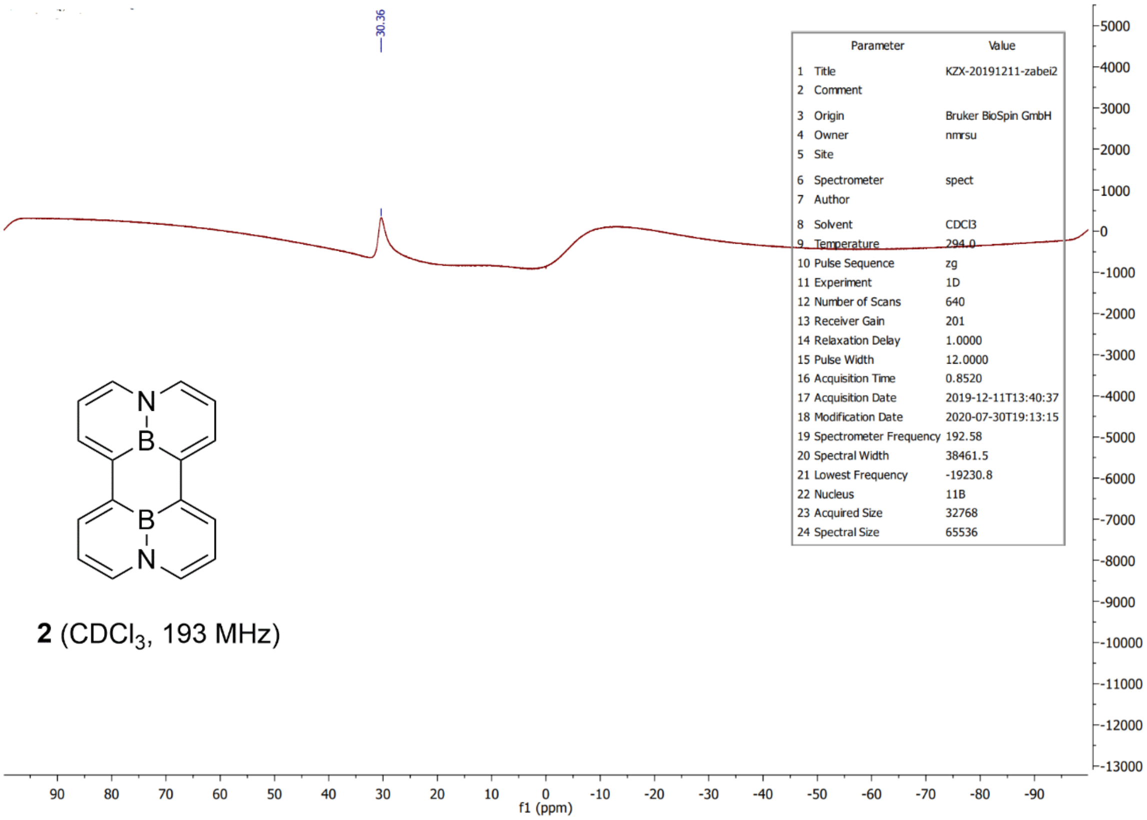

2 ($CDCl_3$, 193 MHz)

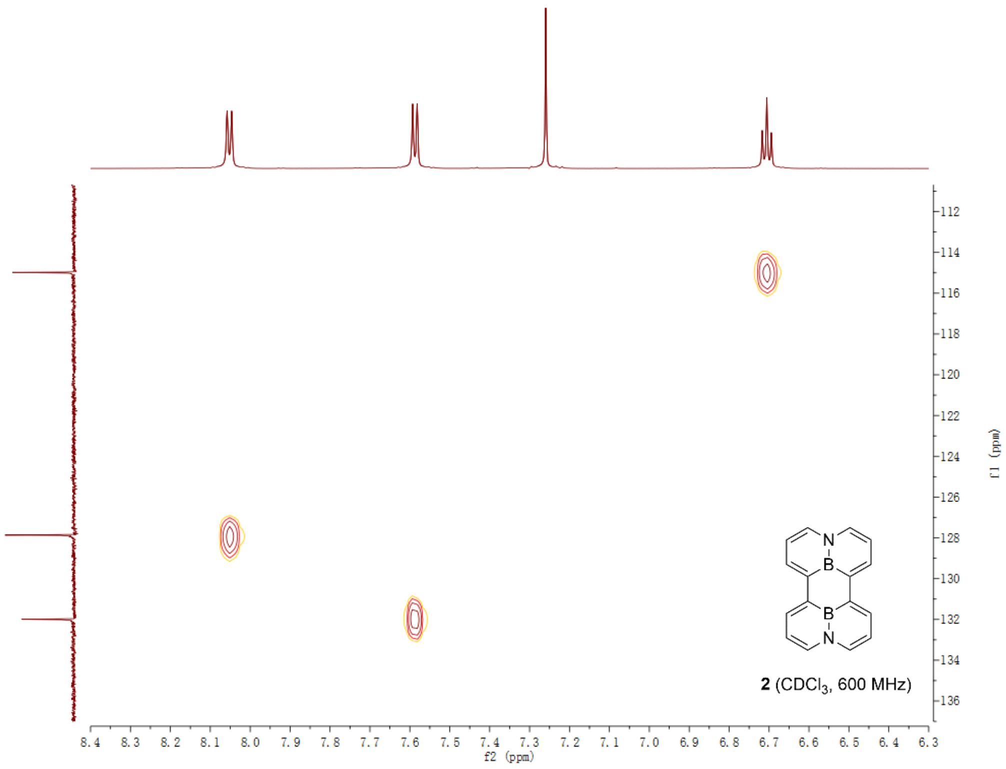

2 ($CDCl_3$, 600 MHz)

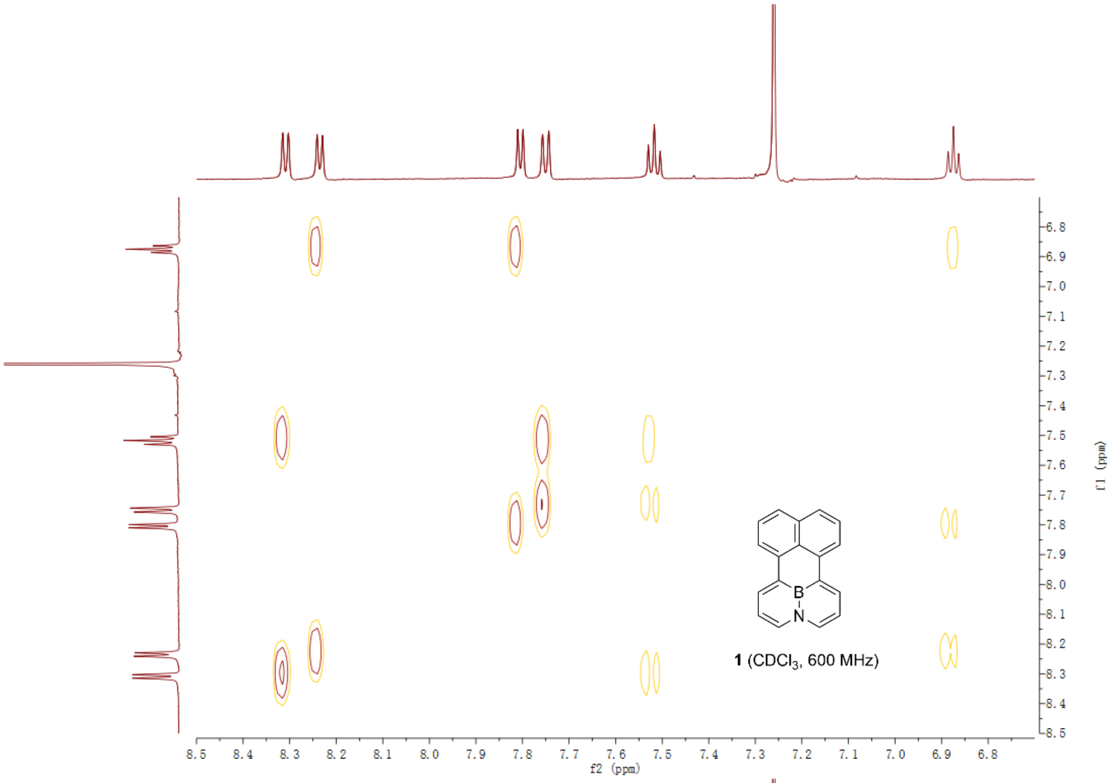
1 (CDCl3, 600 MHz)
f2 (ppm)
f1 (ppm)

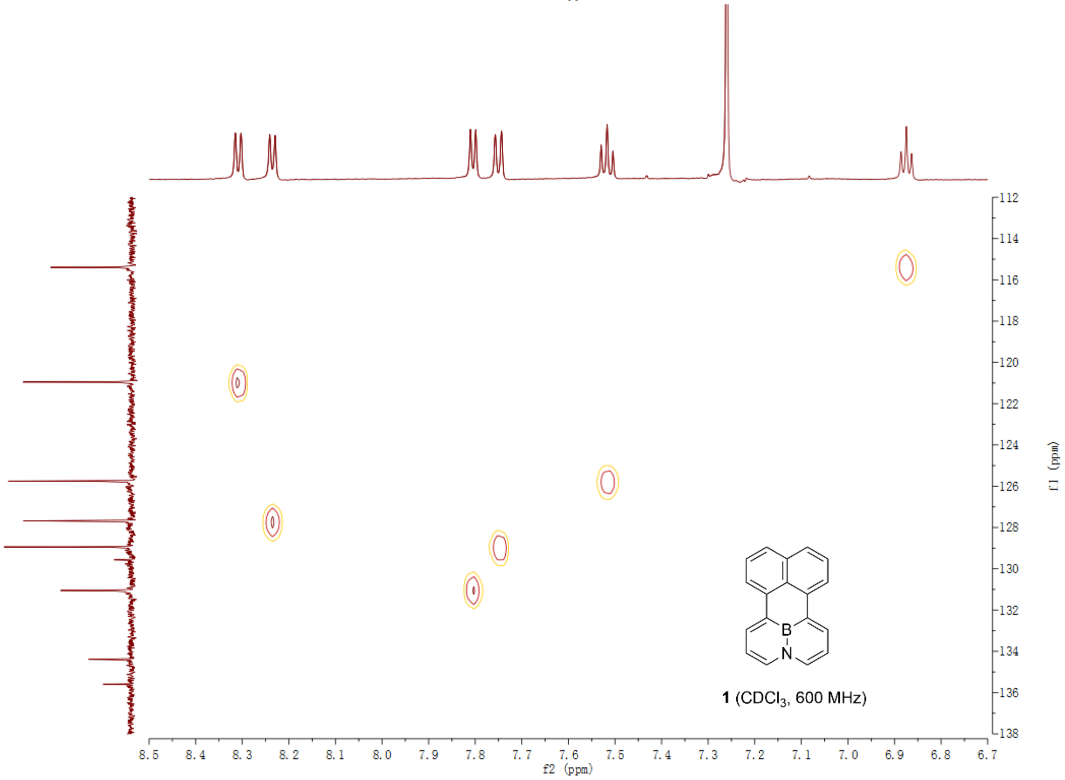
1 (CDCl3, 600 MHz)
f2 (ppm)
f1 (ppm)

File : E:\5973N DATE\2019\201912\1218\Snapshot\H192978.D
Acquired : 18 Dec 2019 12:38
Sample Name : k2x-3
Instrument : Agilent Technologies 5973N
: National Center for Organic Mass Spectrometry in Shanghai, Shanghai Institute of Organic Chemistry

Base m/z:254.2 Inten:565184

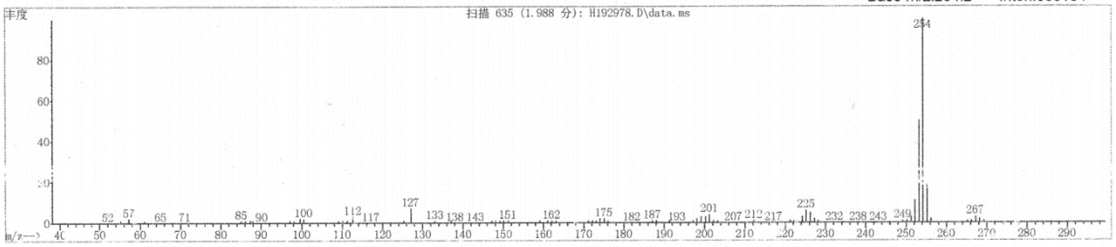

m/z	RA%	m/z	RA%	m/z	RA%	m/z	RA%	m/z	RA%	m/z	RA%	m/z	RA%	m/z	RA%	m/z	RA%
48	0.03	49	0.14	50	0.22	51	0.3	52	0.17	53	0.17	54	0.06	55	0.75	56	0.19
57	2.17	58	0.09	60	0.32	61	0.95	62	0.42	63	0.28	64	0.13	65	0.14	66	0.09
67	0.25	68	0.1	69	0.47	70	0.31	71	0.37	73	0.49	74	0.44	75	0.47	76	0.34
77	0.34	78	0.18	79	0.31	81	0.11	81	0.38	82	0.17	83	0.43	84	0.46	85	1.24
86	1.03	87	1.18	88	0.7	89	0.2	90	0.11	91	0.47	92	0.07	93	0.24	94	0.24
95	0.36	96	0.32	97	0.78	98	0.66	100	1.54	101	1.86	102	0.4	103	0.16	104	0.14
105	0.45	106	0.23	107	0.38	108	0.38	109	0.84	110	0.52	111	0.68	113	2.84	115	0.42
116	0.23	117	0.29	118	0.17	119	0.35	120	0.26	121	0.35	122	0.36	123	0.48	124	0.46
125	0.67	127	7.25	129	0.36	130	0.09	131	0.33	132	0.31	133	0.6	134	0.46	135	0.37
136	0.42	137	0.77	138	0.26	139	0.35	140	0.15	141	0.22	142	0.13	143	0.2	144	0.17
145	0.47	146	0.29	147	1.29	148	0.81	149	1.06	150	0.65	151	0.52	152	0.45	153	0.38
154	0.24	155	0.2	156	0.16	157	0.13	158	0.1	159	0.71	160	0.29	161	0.73	162	0.76
163	0.67	164	0.44	165	0.36	166	0.27	167	0.39	168	0.1	169	0.05	170	0.16	171	0.58
172	0.81	173	1.4	174	1.52	175	2.47	176	1.39	177	0.33	178	0.21	179	0.23	180	0.31
181	0.18	182	0.05	183	0.21	184	0.09	185	0.4	186	0.47	187	0.56	188	0.9	189	0.39
190	0.35	191	1.02	192	0.3	193	0.12	194	0.09	195	0.21	196	0.4	197	0.92	198	1.94
199	3.08	200	2.81	201	4.23	202	1.3	203	0.97	204	0.33	205	0.14	206	0.31	207	0.28
208	0.14	209	0.13	210	0.19	211	0.26	212	0.58	213	0.36	214	0.34	215	0.39	216	0.14
217	0.11	218	0.16	219	0.12	220	0.15	221	0.71	222	0.66	223	1.38	224	3.47	225	6.44
226	5.18	227	2.47	228	0.6	229	0.14	230	0.08	232	0.07	233	0.09	234	0.05	235	0.17
236	0.16	237	0.38	238	0.21	239	0.35	240	0.11	240	0.11	241	0.26	242	0.4	243	0.08
244	0.05	245	0.04	246	0.05	247	0.08	248	0.14	249	0.57	250	1.13	251	2.74	252	10.81
253	49.99	254	100	255	18.63	256	1.64	257	0.21	258	0.1	259	0.12	261	0.12	262	0.08

263	0.11	264	0.38	265	0.58	266	1.31	267	3.05	268	2.01	269	0.6	270	0.34	271	0.23
272	0.05	272	0.06	273	0.11	274	0.05	275	0.14	276	0.06	277	0.08	278	0.11	279	0.14
280	0.2	281	0.34	282	0.24	283	0.16	284	0.19	285	0.04	286	0.08	287	0.06	288	0.07
289	0.03	289	0.06														

8 Peak Value:

254	100	253	49.99	255	18.63	252	10.81	127	7.25	225	6.44	226	5.18	201	4.23

National Center for Organic Mass Spectrometry in Shanghai
Shanghai Institute of Organic Chemistry
Chinese Academic of Sciences
High Resolution MS Data Report

Instrument: Waters Micromass GCT Premier   Ionisation Mode: EI+   Electron Energy: 70eV

Card Serial Number: GCT-P-T19-12-0064

Sample Serial Number: 2011770-20191216-KZX-3

Operator: Li

Date: 2019/12/23

Elemental Composition Report

Single Mass Analysis
Tolerance = 5.0 PPM / DBE: min = -1.5, max = 50.0
Element prediction: Off

Monoisotopic Mass, Odd and Even Electron Ions
349 formula(e) evaluated with 3 results within limits (up to 50 closest results for each mass)
Elements Used:
C: 0-50 H: 0-80 N: 0-2 O: 0-2 F: 0-3 10B: 0-2

Minimum:				-1.5		
Maximum:		2.0	5.0	50.0		
Mass	Calc. Mass	mDa	PPM	DBE	i-FIT	Formula
252.1252	252.1259	-0.7	-2.8	13.0	228.6	C16 H12 N2 10B2
	252.1248	0.4	1.6	5.5	6445.5	C13 H16 O2 F2 10B
	252.1263	-1.1	-4.4	10.0	11105.4	C16 H16 N2 O

File : E:\5973N date\2019\201912\1218\H192977.D
Acquired : 18 Dec 2019 12:21
Sample Name : k2x-2
Instrument : Agilent Technologies 5973N
: National Center for Organic Mass Spectrometry in Shanghai, Shanghai Institute of Organic Chemistry

Base m/z:253.2 Inten:783168

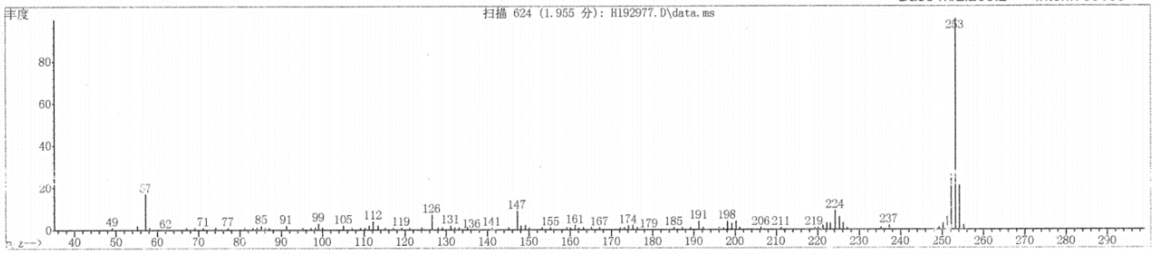

m/z	RA%	m/z	RA%	m/z	RA%	m/z	RA%	m/z	RA%	m/z	RA%	m/z	RA%	m/z	RA%	m/z	RA%
45	0.11	47	0.09	48	0.07	49	0.5	50	0.24	51	0.39	52	0.08	53	0.25	54	0.14
55	2.27	56	0.39	57	16.81	58	0.81	59	0.15	60	0.22	61	0.38	62	0.2	63	0.46
64	0.11	65	0.32	66	0.07	67	0.5	68	0.27	69	1.42	70	0.44	71	1.29	72	0.22
73	0.37	74	0.57	75	0.42	76	0.11	77	0.52	78	0.21	79	0.35	80	0.15	81	0.75
82	0.45	83	1.19	84	0.86	85	1.58	86	1.1	87	1.08	88	0.24	89	0.16	90	0.05
91	2.2	92	0.29	93	0.37	94	0.2	95	0.52	96	0.29	97	1.29	98	1.11	99	2.87
100	1.31	102	0.08	103	0.22	104	0.32	105	1.76	106	0.37	107	0.64	108	0.23	109	0.89
110	0.58	111	2.11	112	3.96	113	1.52	115	0.91	116	0.39	117	1.3	118	0.3	119	1.41
120	0.29	121	0.57	122	0.45	123	0.47	124	0.54	127	7.49	128	0.69	129	0.73	130	0.32
131	1.76	132	0.91	133	1.22	134	0.33	135	0.6	136	0.19	137	0.4	138	0.22	139	0.42
140	0.11	141	0.54	142	0.31	143	0.46	144	0.19	145	0.72	146	0.46	147	8.62	148	1.81
149	1.92	150	0.67	151	0.32	152	0.19	153	0.52	154	0.24	155	0.59	156	0.14	157	0.32
158	0.24	159	1.42	160	0.51	161	1.87	162	0.53	163	1.37	164	0.26	165	0.67	166	0.17
167	0.67	168	0.16	169	0.27	170	0.13	171	0.41	172	1.03	173	0.91	174	1.84	175	2.2
176	0.56	177	0.36	178	0.35	179	0.26	180	0.15	181	0.2	182	0.15	183	0.3	184	0.19
185	0.66	186	0.45	187	1.08	188	0.42	189	1.35	190	0.48	191	4.16	192	0.78	193	0.28
194	0.22	195	0.29	196	0.72	197	1.49	198	3.89	199	2.65	200	3.67	201	1.18	202	0.41
203	0.26	204	0.19	205	0.43	206	0.53	207	0.32	208	0.3	209	0.33	210	0.18	211	0.53
212	0.33	213	0.29	214	0.28	215	0.19	216	0.09	217	0.11	218	0.13	219	1.01	220	0.63
221	1.5	222	2.54	223	3.16	224	9.06	225	6.06	226	2.7	227	1.03	228	0.12	229	0.16
230	0.06	231	0.05	232	0.03	233	0.16	235	0.58	236	0.2	237	1.67	238	0.36	239	0.29
240	0.05	241	0.04	241	0.06	242	0.09	243	0.04	244	0.05	245	0.05	246	0.08	247	0.21
248	0.42	249	1.41	250	3.13	251	5.69	252	25.94	253	100	254	21.47	255	2.26	256	0.2
257	0.07	258	0.03	259	0.07	260	0.03	261	0.25	262	0.13	263	0.1	264	0.12	265	0.18
266	0.13	267	0.2	268	0.07	269	0.11	271	0.08	272	0.03	273	0.17	274	0.09	275	0.08
277	0.07	278	0.06	279	0.23	280	0.18	281	0.23	282	0.06	283	0.14	284	0.1	285	0.28
286	0.12	287	0.22	288	0.09	288	0.08	289	0.32								

8 Peak Value:

253	100	252	25.94	254	21.47	57	16.81	224	9.06	147	8.62	127	7.49	225	6.06

National Center for Organic Mass Spectrometry in Shanghai
Shanghai Institute of Organic Chemistry
Chinese Academic of Sciences
High Resolution MS Data Report

Instrument: Waters Micromass GCT Premier Ionisation Mode: EI+ Electron Energy: 70eV

Card Serial Number: GCT-P-T*19-12*-0065

Sample Serial Number: 2011770-20191216-KZX-2

Operator: Li

Date: 2019/12/23

Elemental Composition Report

Single Mass Analysis
Tolerance = 5.0 PPM / DBE: min = -1.5, max = 50.0
Element prediction: Off

Monoisotopic Mass, Odd and Even Electron Ions
349 formula(e) evaluated with 2 results within limits (up to 50 closest results for each mass)
Elements Used:
C: 0-50 H: 0-80 N: 0-2 O: 0-2 F: 0-3 10B: 0-2

Minimum:				-1.5		
Maximum:		2.0	5.0	50.0		
Mass	Calc. Mass	mDa	PPM	DBE	i-FIT	Formula
252.1102	252.1099	0.3	1.2	14.0	16.0	C18 H12 N 10B
	252.1111	-0.9	-3.6	10.0	15.5	C15 H13 N O F 10B

File : E:\5973N DATE\2019\201912\1218\Snapshot\H192976.D
Acquired : 18 Dec 2019 12:18
Sample Name : k2x-1
Instrument : Agilent Technologies 5973N
: National Center for Organic Mass Spectrometry in Shanghai, Shanghai Institute of Organic Chemistry

Base m/z:254.2 Inten:623424

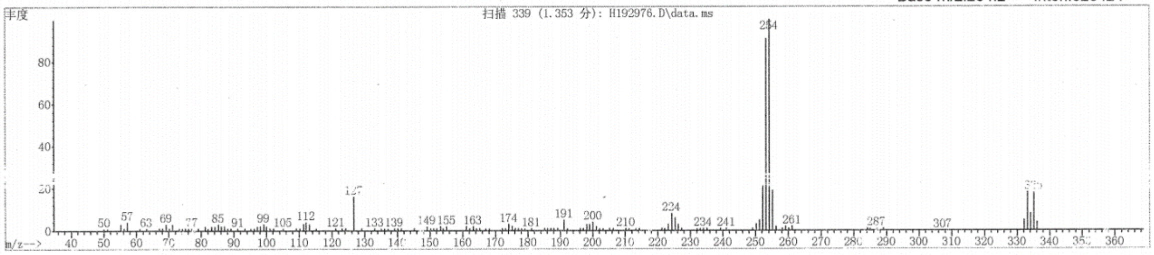

m/z	RA%	m/z	RA%	m/z	RA%	m/z	RA%	m/z	RA%	m/z	RA%	m/z	RA%	m/z	RA%	m/z	RA%
45	0.11	47	0.14	48	0.07	49	0.4	50	0.52	51	0.68	52	0.28	53	0.25	54	0.23
55	2.66	56	1.17	57	4.25	58	0.32	59	0.39	60	0.44	61	0.77	62	0.45	63	0.69
64	0.23	65	0.32	66	0.1[illegible]	67	1.04	68	0.55	69	2.54	70	1.21	71	2.6	72	0.39
73	0.96	74	1.33	75	0.99	76	0.59	77	1.11	78	0.23	79	0.56	80	0.17	80	0.17
81	1.55	82	0.84	83	2.22	84	2.02	85	2.91	86	1.61	87	1.97	88	0.52	89	0.52
91	0.86	92	0.24	93	0.63	95	0.9	96	0.71	97	2.32	98	1.83	99	3.41	100	2.39
101	0.81	102	0.62	103	0.31	104	0.22	105	0.84	106	0.38	107	0.33	108	0.34	109	0.99
110	0.66	111	2.52	112	4.49	113	3.02	115	0.98	116	0.32	117	0.44	118	0.1	119	0.38
120	0.23	121	0.75	122	0.49	123	0.71	124	0.8	127	16.48	129	0.54	130	0.22	131	0.39
132	0.16	133	0.58	134	0.35	135	1.1	136	0.6	137	0.99	138	0.46	139	0.94	140	0.52
141	0.59	142	0.17	143	0.31	144	0.24	145	0.64	146	0.28	147	0.68	148	1.17	149	2.4
150	1.46	151	1.01	152	1.45	153	1.82	154	1.42	155	1.64	156	0.29	157	0.24	158	0.24
159	0.29	160	0.45	161	1.95	162	0.71	163	1.54	164	0.5	165	0.89	166	0.49	167	0.81
168	0.6	169	0.48	170	0.4	171	0.36	172	0.8	173	1.32	174	2.54	175	1.96	176	1.08
177	0.75	178	0.74	179	0.56	180	0.37	181	0.61	182	0.45	183	0.49	184	0.31	185	0.75
186	0.58	187	1.25	188	0.74	189	0.62	190	0.31	191	5.28	192	0.95	193	0.27	194	0.14
195	0.42	196	0.6	197	1.35	198	3.4	199	2.8	200	3.85	201	1.5	202	0.88	203	0.61
204	0.35	205	0.54	206	1.13	207	0.39	208	0.44	209	0.32	210	0.53	211	0.84	212	0.48
213	0.58	214	0.79	215	0.34	216	0.17	217	0.24	218	0.11	219	0.29	220	0.46	221	0.62
222	1.49	223	2.78	224	8.42	225	5.52	226	3.23	227	1.43	228	0.48	229	0.15	230	0.1
231	0.19	232	0.58	233	0.58	234	0.66	235	0.55	236	0.14	237	0.33	238	0.38	239	0.51
240	0.31	241	0.67	242	0.23	243	0.42	244	0.15	245	0.18	246	0.05	247	0.18	248	0.49
249	1.46	250	3.17	251	5.32	252	20.69	253	91.07	254	100	255	18.76	256	2.13	257	0.38

258	0.56	259	2.1	260	0.81	261	2.09	262	0.35	263	0.2	264	0.19	265	0.08	266
267	0.1	268	0.16	269	0.13	270	0.27	271	0.05	272	0.04	273	0.27	274	0.22	275
276	0.22	277	0.39	278	0.18	279	0.13	280	0.3	281	0.36	282	0.23	284	0.6	285
286	0.43	287	1.29	288	0.37	289	1.16	290	0.28	291	0.17	292	0.15	293	0.1	293
294	0.09	295	0.07	295	0.2	296	0.07	297	0.06	299	0.03	301	0.04	302	0.04	303
304	0.06	305	0.09	306	0.04	306	0.05	307	0.08	308	0.2	309	0.29	310	0.14	311
313	0.05	314	0.1	316	0.15	317	0.12	318	0.05	319	0.11	320	0.05	321	0.07	323
323	0.05	324	0.11	324	0.05	325	0.05	327	0.17	328	0.07	329	0.1	330	0.05	331
332	4.96	333	18.21	334	8.2	335	17.73	336	3.51	337	0.43	338	0.07	339	0.14	340
341	0.27	343	0.04	344	0.05	349	0.03	349	0.04	350	0.05	351	0.03	351	0.11	352
353	0.08	353	0.07	354	0.09	355	0.06	356	0.13	357	0.06	358	0.05	358	0.04	

8 Peak Value:

254	100	253	91.07	252	20.69	255	18.76	333	18.21	335	17.73	127	16.48	224	8.42

National Center for Organic Mass Spectrometry in Shanghai
Shanghai Institute of Organic Chemistry
Chinese Academic of Sciences
High Resolution MS Data Report

Instrument: Waters Micromass GCT Premier   Ionisation Mode: EI+   Electron Energy: 70eV

Card Serial Number: GCT-P-T19-12-0066

Sample Serial Number: 2011770-20191216-KZX-1

Operator: Li

Date: 2019/12/23

Elemental Composition Report

Single Mass Analysis
Tolerance = 5.0 PPM / DBE: min = -1.5, max = 50.0
Element prediction: Off

Monoisotopic Mass, Odd and Even Electron Ions
355 formula(e) evaluated with 4 results within limits (up to 50 closest results for each mass)
Elements Used:
C: 0-50 H: 0-80 N: 0-2 O: 0-4 Br: 0-1 10B: 0-2

Minimum:				-1.5		
Maximum:		2.0	5.0	50.0		
Mass	Calc. Mass	mDa	PPM	DBE	i-FIT	Formula
332.0370	332.0361	0.9	2.7	13.0	2.7	C18 H13 N Br 10B
	332.0368	0.2	0.6	8.0	1070.9	C12 H13 N2 O3 Br 10B2
	332.0372	-0.2	-0.6	5.0	1347.5	C12 H17 N2 O4 Br
	332.0374	-0.4	-1.2	25.0	1727.0	C25 H4 N2
